# Correcting spelling mistakes in Persian texts with rules and deep learning methods

**DOI:** 10.1038/s41598-023-47295-2

**Published:** 2023-11-15

**Authors:** Sa. Kasmaiee, Si. Kasmaiee, M. Homayounpour

**Affiliations:** Department of Computer Engineering, Amirkabir University of Technology, Tehran, Iran

**Keywords:** Computational science, Computer science, Information technology

## Abstract

This study aims to develop a system for automatically correcting spelling errors in Persian texts using two approaches: one that relies on rules and a common spelling mistake list and another that uses a deep neural network. The list of 700 common misspellings was compiled, and a database of 55,000 common Persian words was used to identify spelling errors in the rule-based approach. 112 rules were implemented for spelling correction, each providing suggested words for misspelled words. 2500 sentences were used for evaluation, with the word with the shortest Levenshtein distance selected for evaluation. In the deep learning approach, a deep encoder-decoder network that utilized long short-term memory (LSTM) with a word embedding layer was used as the base network, with FastText chosen as the word embedding layer. The base network was enhanced by adding convolutional and capsule layers. A database of 1.2 million sentences was created, with 800,000 for training, 200,000 for testing, and 200,000 for evaluation. The results showed that the network's performance with capsule and convolutional layers was similar to that of the base network. The network performed well in evaluation, achieving accuracy, precision, recall, F-measure, and bilingual evaluation understudy (Bleu) scores of 87%, 70%, 89%, 78%, and 84%, respectively.

## Introduction

Natural Language Processing (NLP) is a field that bridges the gap between human language and computers. This technology enables applications to comprehend, procedure, and interpret human language. The significance of NLP as a tool for comprehending information generated by humans stems from the fact that data depends on the context^1^. NLP encompasses a wide range of topics related to the computational processing and understanding of human languages.

Since the 1980s, data-driven methods such as statistics, probability, and machine learning have become increasingly popular in this field. Recent advances in computing power and the parallelization of graphics processing units have enabled the use of deep learning techniques. Deep neural networks with millions of trainable parameters are used in this learning process. The availability of large datasets collected through complex processes makes it possible to train such deep architectures^1,2^.

The growth of text data in recent years highlights the importance of text processors. Research in this field is ongoing because text-based applications have not performed satisfactorily despite their long history and current developments. Text processing is more challenging than processing data such as images, speech, videos, etc., due to complexities such as metaphors, homographs, etc.^3^. Text-based applications are numerous and include information retrieval^4–7^, document classification and sentiment analysis^8–12^, keyword extraction^13^, word embedding^14^, handwriting recognition^15^, etc. In the fields of education and medicine, deep learning is of great interest to researchers due to its capabilities and the creation of suitable platforms^16–18^.

Spelling is a component of word processing that checks the correctness of words and makes necessary corrections if there are errors. This task is one of the simplest applications that involves text processing. Many research studies have been conducted on the automatic correction of misspellings in several languages, especially English, and there are powerful tools available in this field^19–21^. However, there is a lack of research and powerful tools in this field for the Persian language.

In this study, an attempt has been made to identify and correct the misspelled word. This study was the first to conduct a comprehensive investigation of spelling correction in Persian texts using deep learning and a rule-based. A training database was created using the rules approach. According to other studies conducted in Persian language processing, 112 rules were devised for spelling correction and applied to Persian texts in this study. It is discussed in detail in the Rules for Text Correction section. A database containing 1.2 million sentences was obtained. The capsule layer, FastText embedding, and CNN in LSTM encoder decoder were employed for the first time in Persian text error correction and their impact was examined. A graphical interface-based system was developed with Django^22^. The results indicated that the designed system had high accuracy in identifying and correcting errors in the Persian language. The background of the research and related studies will be discussed first, followed by the method for detecting and correcting spelling errors using two approaches based on rules and deep networks. Finally, an evaluation of these two approaches and their comparison will be presented.

## Related work

**Figure 1:**
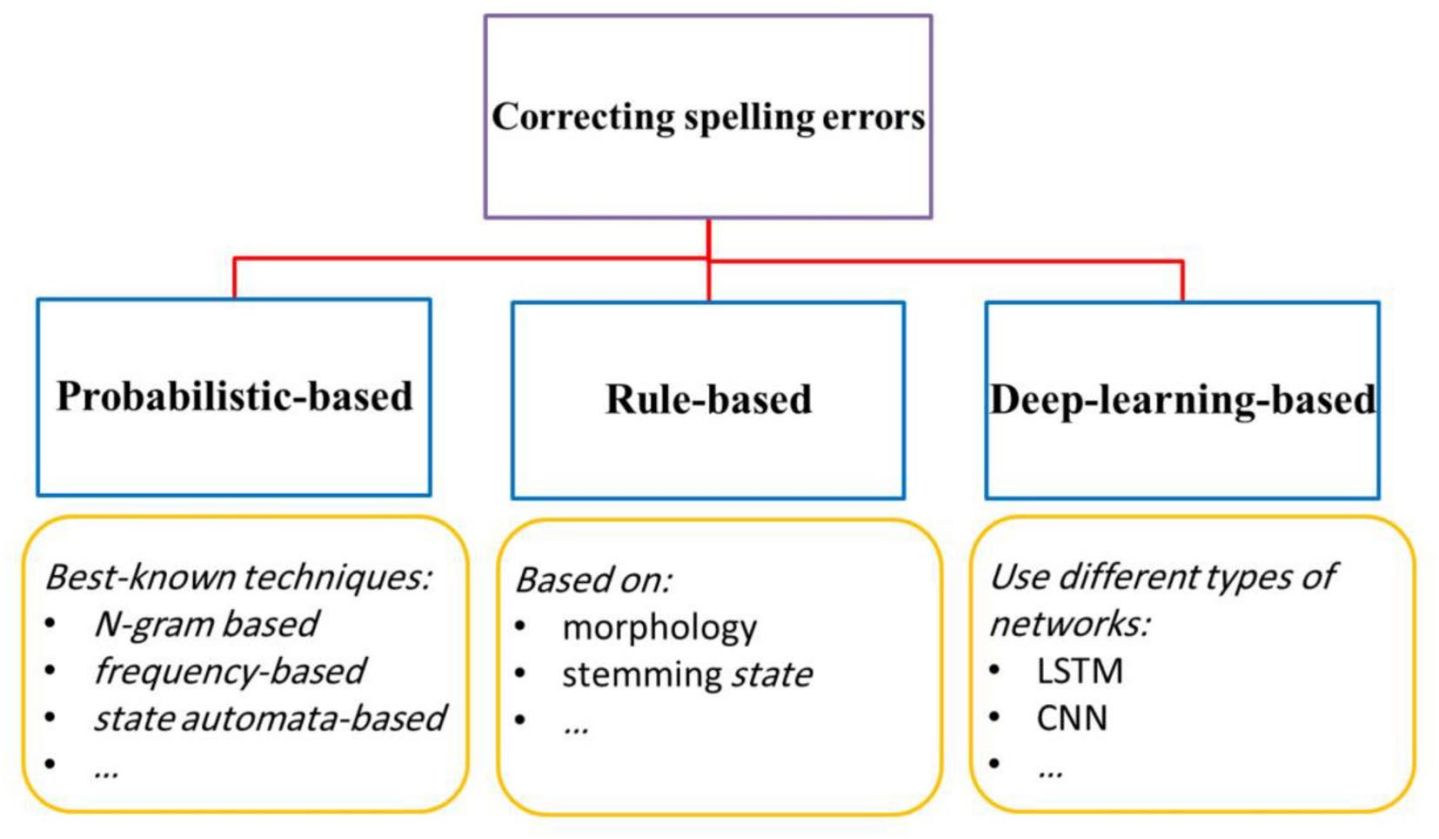
Types of spelling correction techniques in literature.

Spell correction is a field of research within NLP that has been extensively studied. Numerous spelling correction techniques have been developed for various languages spoken worldwide. This section examines studies related to the topic of this research. As shown in Fig. 1, spelling correction techniques based on previous literature include probabilistic-based, rule-based, and deep-learning-based approaches. These three approaches are discussed in the following sections.

### Probabilistic and rule-based methods

This category includes methods that do not need any specific language knowledge. The most well-known techniques in this group are n-gram-based, frequency-based, and finite state automata-based. These techniques rely on the counts and features of the words. N-gram-based techniques operate based on unigram, bi-gram, tri-gram, etc. This method splits each text string into a set of adjacent n-grams^23,24^. In this technique, the error probability of each n-gram should be calculated individually. This method can detect the position of the misspelled words with or without a dictionary. The word-frequency method identifies and corrects errors by using the probability of common mistakes repetition and the distance-editing method^25,26^. Singh et al.^26^ proposed a spelling checker based on frequency and a grammar checker based on rules. They used a dictionary and bigram to find spelling errors and chose the best word for spelling correction. In the grammar part, they applied rules that can determine the grammaticality of the sentences based on the verb tense and whether it is negative or positive. In the finite state machine, the computational model of mathematics is used to design natural language. A word accepted by the finite state machine model is considered correct in the language^27,28^. The limitation of these methods is that some spelling errors require specific language knowledge, and these methods cannot detect and correct such errors because they employ features such as frequency, word count, and others.

Rules use heuristics derived from morphology, stemming, and other features of words to correct the spelling. These rules usually correct errors with inserted and deleted letters. Despite the recent advances in applying deep neural networks such as sequence-to-sequence language modeling, rule-based methods are still used for morphologically rich languages like Kurdish due to accuracy and feasibility^29^. Such rules generally work based on probabilities^30^. An improved Morphological Analyzer (MA) has been developed to enhance the Cushitic grammar checker tool^31^ and Persian Optical Character Recognition^32^. In the Persian language, some spelling structures can be expressed as rules and studies have been done on this topic^33,34^. The main disadvantage of these methods is the need to explore different heuristic rules for each language. Therefore, rules related to a specific language are not used for other languages. The performance of the error corrector can be enhanced by combining several methods. Aziz et al.^35^ could find wrong spellings of an Urdu word using a broad lexicon lookup method and provide a list of candidate words with correct spellings. They used a mixed model that rates the words in the nominee word list. They used several ranking techniques such as Soundex^36^, Shapex^36^, longest common subsequence (LCS)^37^, and N-gram, as well as their combinations, to find the finest technique in terms of F1 score.

### Deep‑learning‑based method

In many cases, we need to know and understand the word context in relation to sentence to recognize the error. In these cases, the deep network is helpful. Kaur et al.^38^ used the tree data structure to store the dictionary. They used a recurrent neural network that incorporates Long-short term memory (LSTM) unit to reduce the vector distance between the error word and the word in the dictionary to correct errors. They also applied rules that were manually crafted, rules that followed the syntax of the language, and rules that dealt with tokenization to enhance the detection and correction of errors. The error detection method was the absence of words in the database after pre-processing, and the error correction was done by n-gram and rules. Finally, if it was not found in the database, the network suggested the closest sentence. A deep learning-based spell checker for Malayalam was proposed by Sooraj et al.^39^. They trained a neural network that uses LSTM to detect misspelled words and the location of the errors in the error detection phase. They used the F measure to evaluate the accuracy of error detection and they selected the most probable word from the candidate words to correct the errors. Caryappa et al.^40^ developed a deep learning model that incorporates LSTM units that could detect grammatical errors in the Dravidian language. They implemented neural network-based word embedding in their model.

Hu et al.^41^ used the combination of encoder representations from transformers (BERT) and the algorithm of Levenshtein editing distance to rank and choose the word that corrects the spelling. They showed that if these two are appropriately combined, spelling errors will be edited effectively, although they only examined the accuracy criterion in their study. Beloki et al.^42^ employed neural models of sequence-to-sequence transformation to correct grammatical errors in the Basque language. They did not have any training data for this task, so with a rule-based method, they created grammatically incorrect sentences from a set of terms extracted from 500,000 news stories, used them for training and obtained a network with an F criterion of 0.87. Zolzaya et al.^43^ conducted the first text normalization study for the Mongolian language. Their goal was to convert the text with errors in social networks into an official one. The original texts were Roman and had to be converted to Cyrillic. They used a two-stage model; the first stage included a neural network to convert sequence to sequence at the character level and its output as input in the second stage—the second stage combined two methods based on LSTM and the editing algorithm. Mandal and Nanmaran^44^ presented a new architecture focusing on type normalization. One of the main features of their architecture was that, in addition to normalization, it could be applied to restore text and generate keyword.

Mager et al.^45^ normalized social texts with colloquial errors. Using a sequence-to-sequence neural network, they obtained a 5% improvement in results compared to the previous model. Singh and Singh^46^ corrected spelling errors in Hindi using deep learning. In this process, the misspelled words were automatically identified by the network and replaced by the closest word. They used a database containing 9 gigabytes of data to train and test their network. They implemented two scenarios to test their neural network. They reported an accuracy of 83%, recall of 72%, and F-measure of 76% as the network results for the test data. Some studies^47,48^ have examined the combined effect of rules and neural network-based methods, which shows an improvement in the accuracy of the hybrid model. Sampath and Shanmugavel^47^ suggested a hybrid approach that combines the algorithm of Levenshtein’s edit distance, the algorithm based on rules, the algorithm of Soundex, and the model of LSTM for the spell checker of the Tamil language. Their proposed machine had an accuracy of 95.67% and a Tamil scholar verified it.

### Ethical and informed consent for data used

This paper was done by the authors, and no human participants other than the authors were involved in it, and informed consent was obtained from all authors.

## Background

### Artificial neural networks

**Figure 2:**
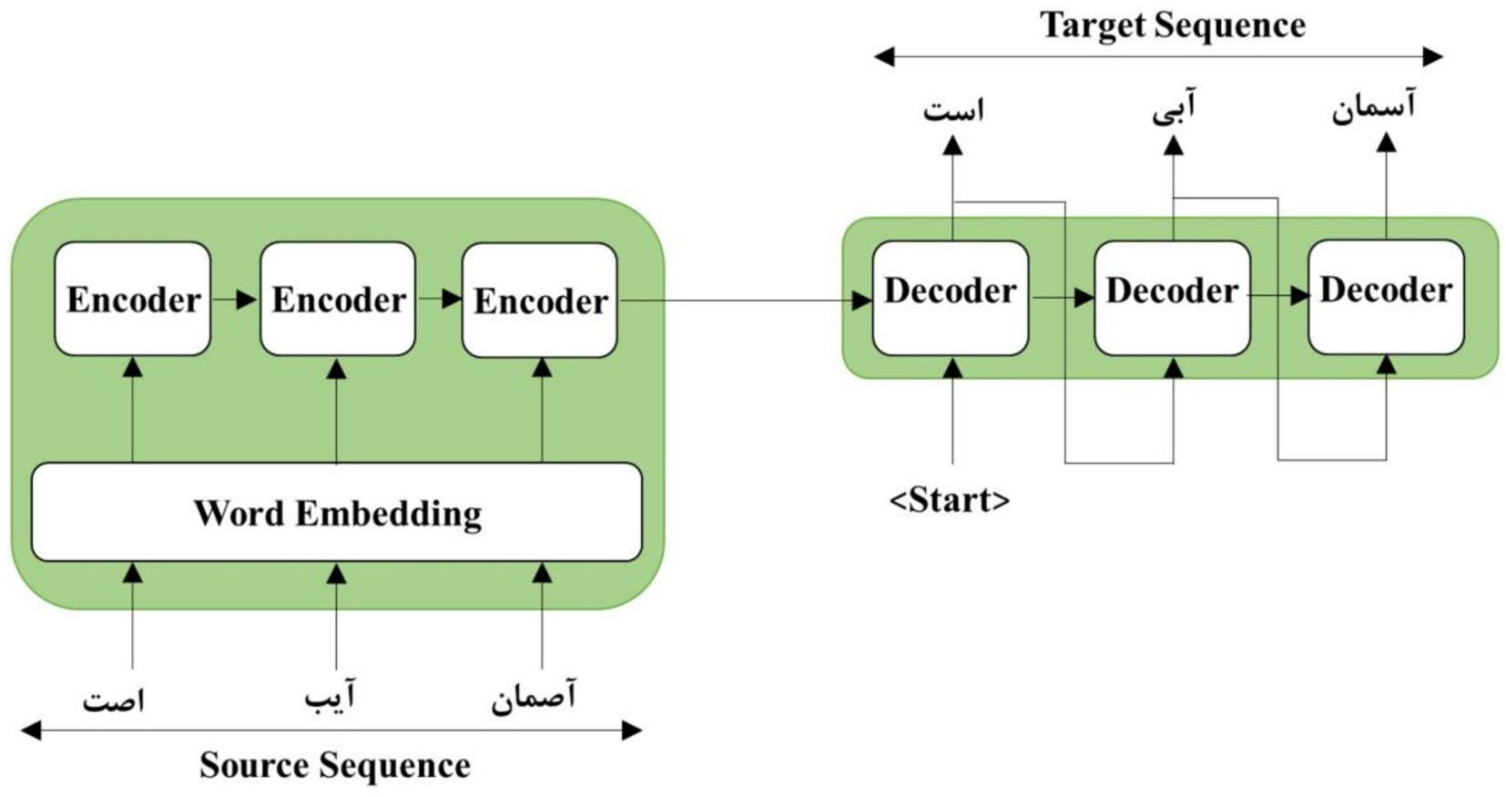
Seq2Seq framework.

Artificial neural networks (ANNs) are computational models inspired by the human brain and other organisms^49^. The learning procedure of neural networks can be accomplished in three methods: supervised, unsupervised, and reinforced, which depend on the type of network^49^. Neural networks are applied in the automatic correction of texts in two main parts: word representation and vocabulary correction. Different types of networks can be applied in each part to improve performance. Most basic frameworks in natural language processing programs depend on sequence-to-sequence models in which both input and output are sequences. These models are used for different tasks that involve natural language processing, such as translating languages, text summarization, speech-to-text conversion, and text-to-speech conversion. The most common sequence-to-sequence framework has an encoder and a decoder. An example of this framework is shown in Fig. 2. The input data sequence is processed by the encoder, which generates an intermediate output. This output is then used by the decoder to create a series of final outputs^50^. With the development of neural networks, their usage to improve the sequence-to-sequence process was considered^49,50^. The rest of this section mentions the neural networks used in this research. The advantages and reasons for using them are discussed in each part.

### Encoder

An encoder takes a sequence of input data and produces an output at an intermediate level. Different types of networks, such as CNN, capsule, RNN, LSTM, and Bidirectional are used for different purposes. The kinds of neural networks that can be useful in this research are described below^1,3,51–67^.

#### Neural network for vocabulary representation

**Figure 3:**
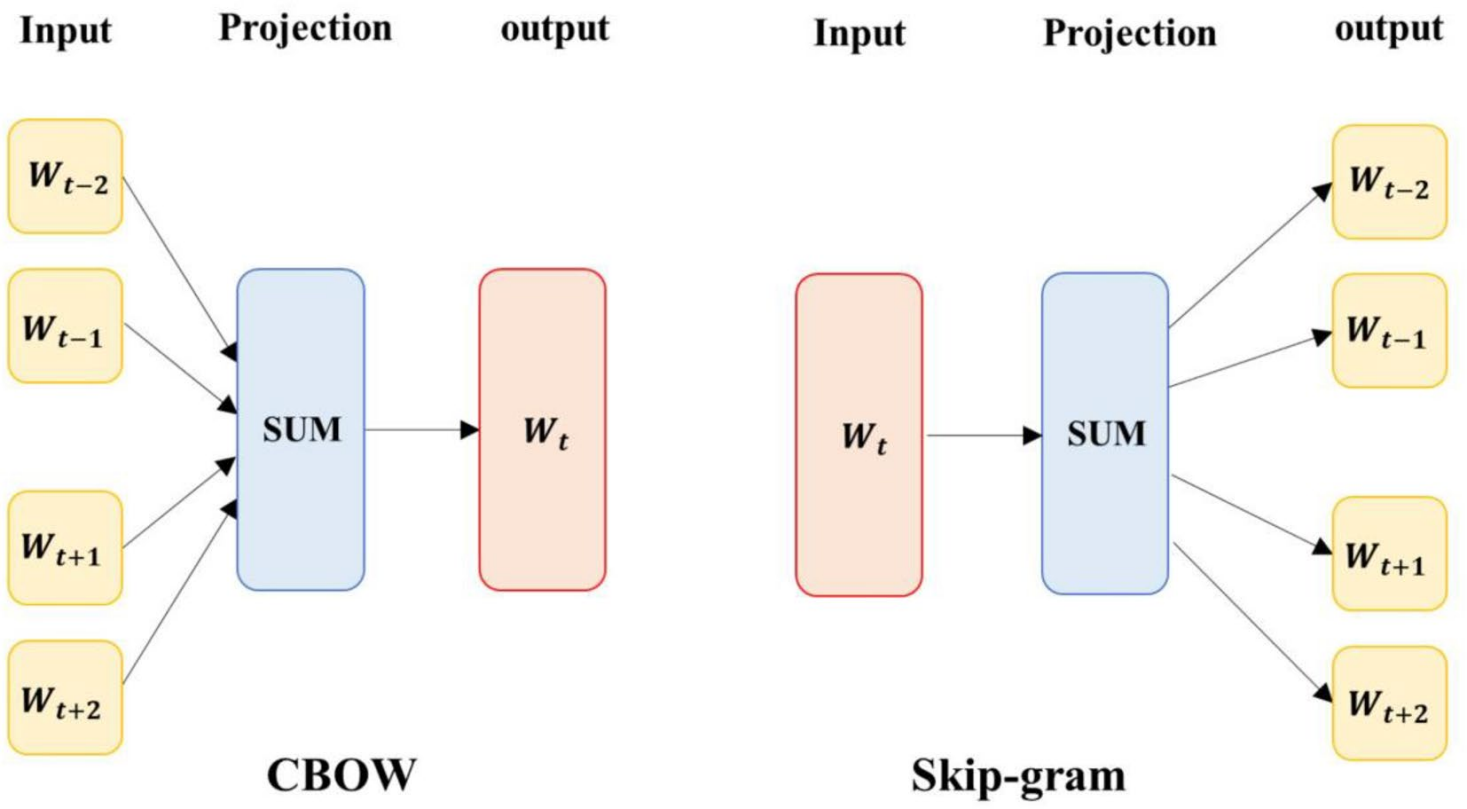
CBOW and Skip-Gram models.

Word embedding is a representation of words as numerical vectors. In fact, by embedding words, vocabulary is mapped to a vector space that different language models can learn. To use the neural network to represent the words, the word-to-vector mapping algorithm is coupled with the learning algorithm. These networks are of the shallow type and are trained for this purpose and typically have between 50 and 300 dimensions^3^. Two common algorithms CBOW and Skip-Gram are used for word embedding. The CBOW model predicts the target word from the surrounding words, while the Skip-Gram model predicts the surrounding words from the target word^51,52^. The general principle of these two models is presented in Fig. 3. The purpose of mapping words to vector space is to group similar words in this space. The following relation determines the criterion of similarity^52^:

1 $$\mathrm{cos}\left({vec}_{i},{vec}_{j}\right)=\frac{{vec}_{i}{vec}_{j}}{\Vert {vec}_{i}\Vert  \Vert {vec}_{j}\Vert }$$

#### Neural network for applying various filters, removing noise, and extracting main features

**Figure 4:**
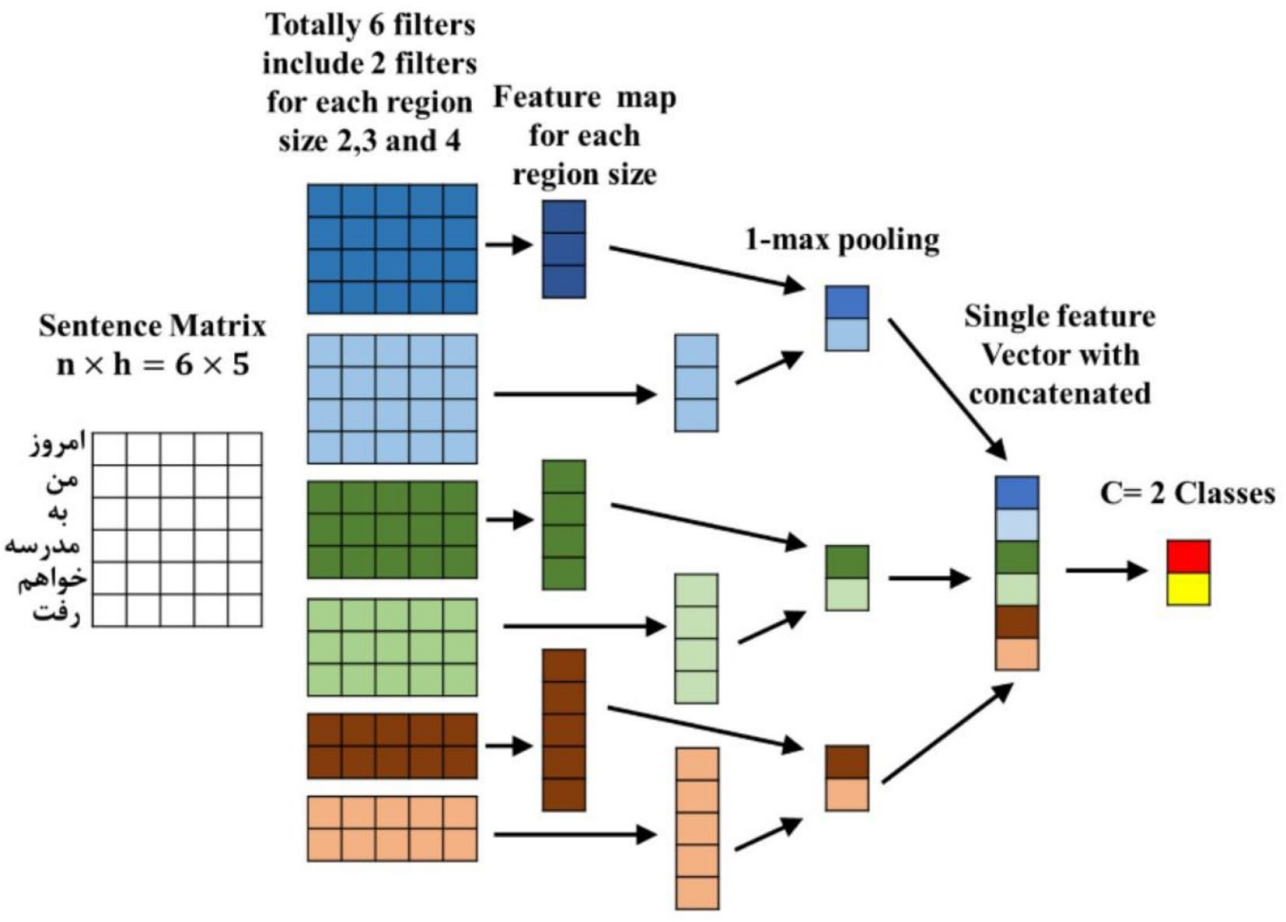
CNN architecture.

Because of their structure, Convolutional networks can apply different kinds of filters on the input. These filters help them to identify the main patterns in the input data. A convolutional neural network includes an input layer, an output layer, and several hidden layers. The hidden layers can be of different types, such as convolutional, pooling, or fully connected. The convolutional layers perform convolutions on the inputs. CNNs use pooling layers that reduce the number of neurons in one layer by combining the outputs of several neurons into one neuron in the next layer. For instance, the max pooling method selects the highest value from a group of neurons in the previous layer and passes it to the next layer. The pooling layer’s task is to reduce the matrix’s dimensions to extract the main features and reduce the computational cost. Fully connected layers are like a multilayer perceptron responsible for mapping^53–55^. Different parts of this network are shown in Fig. 4. Assuming that the sentence length and size of the word window are n and h, respectively. The $c\in {\mathbb{R}}^{n-h+1}$ feature vector is obtained by applying the convolution and pooling layers on the input sequence from the following relations^53^:

2 $${c}_{i}=f\left(w{x}_{i:i+h}+b\right)$$

3 $$\widehat{c}=\mathrm{max}(c)$$

where $w\in {\mathbb{R}}^{hk}$ is the filter and $b$ is a term that adds a constant value to the output. ${x}_{i:j}$ represents the combination of words $i$ to $j$. $f$ is also an activation function, which is usually considered as $tanh$.

#### Neural network for maintaining hierarchical relationships

A capsule neural network is a type of ANN that can model hierarchical relations better. This network adds structures called capsules to the convolutional neural networks to keep the information about the hierarchy. In multilayer perceptron networks, each neuron’s output is a scalar. In convolutional networks, each number obtained is a convolution between a kernel and a part of the input data. The convolutional layer produces the output by stacking these matrices on top of each other, which are the results of applying filters to the input image. Then, the pooling is applied to these matrices. In this network, a lot of useful input data is lost. To address this problem, a capsule network is used. In capsule networks, all the crucial features are conserved and stored as a vector, which has size, direction, and position properties. They can be used to learn hierarchical relations^56^.

#### Neural network for encoding wrong words

**Figure 5:**
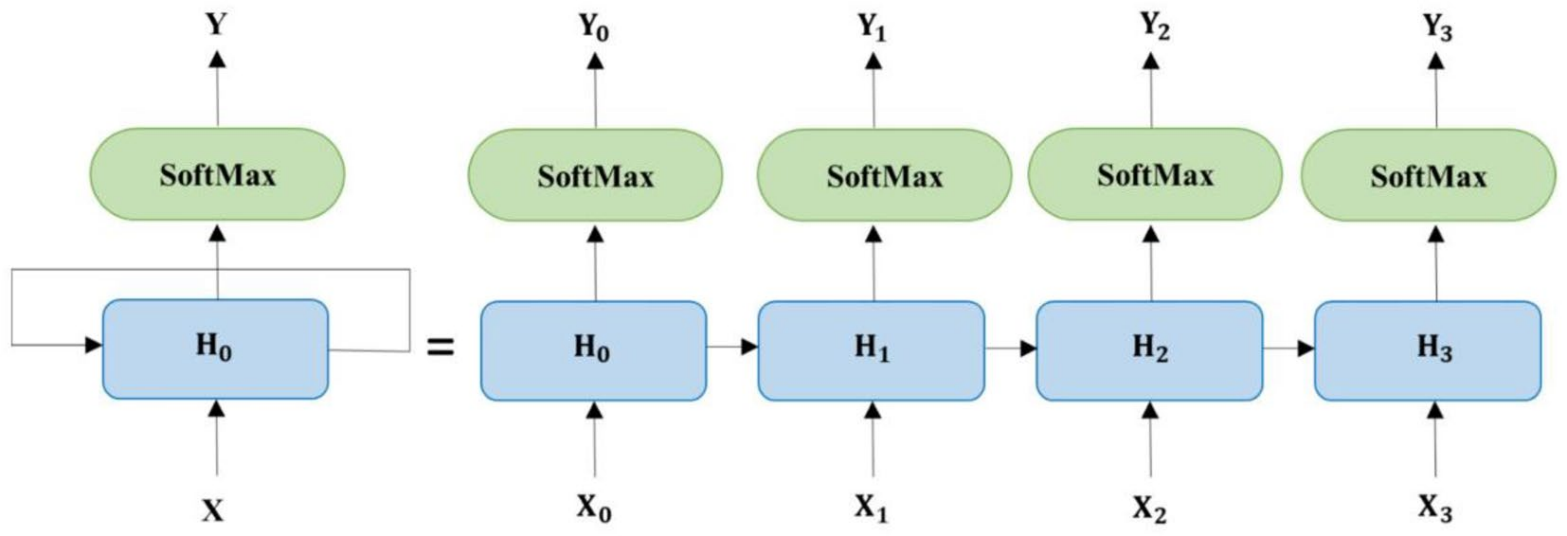
RNN architecture.

A recurrent neural network (RNN) is a type of ANN that can process sequential data by connecting a series of feed-forward networks and passing the output of each network as input to the next one. A recurrent network has three types of layers like feed-forward neural networks. In sequential models, the network receives one vector at each time step from the input vectors. The network processes the input batch and updates its weights accordingly, and then takes the next input batch to continue the operation. Therefore, at each time step, the network uses not only the input vector at that time but also the variables of the previous hidden layer as inputs to the current stage. RNNs have hidden layers that can act as memory by storing information from previous inputs. This feature makes them very suitable for applications that deal with sequential inputs, such as language modeling. In NLP and machine translation, sequences are taken from inputs, and sequences are produced from outputs^1^. Figure 5 shows an illustration of it. Let ${x}_{t}$, ${v}_{t}$,and ${h}_{t}$ represent the input vector, the current word, and its memory, respectively. Then we have^57^:

4 $${x}_{t}={v}_{t}+{h}_{t-1}$$

Assuming that ${w}_{t}$, ${u}_{t}$, and ${b}_{t}$ represent the input vector’s weight matrix, the recurrent weight matrix, and the bias, respectively^57^:

5 $${h}_{t}=\mathrm{tanh}({w}_{t}{x}_{t}+{u}_{t-1}{h}_{t-1}+{b}_{t})$$

The output of the network is obtained from the following relation^58^:

6 $${y}_{t}=\mathrm{Softmax}({u}_{t}{h}_{t})$$

where the Softmax function maps the output vector to the possible output word. The recurrent network problem is that their ability to remember and use the information they learned from the far past diminishes as the sequence gets longer. In other words, they cannot use information from the far past^57,58^.

**Figure 6:**
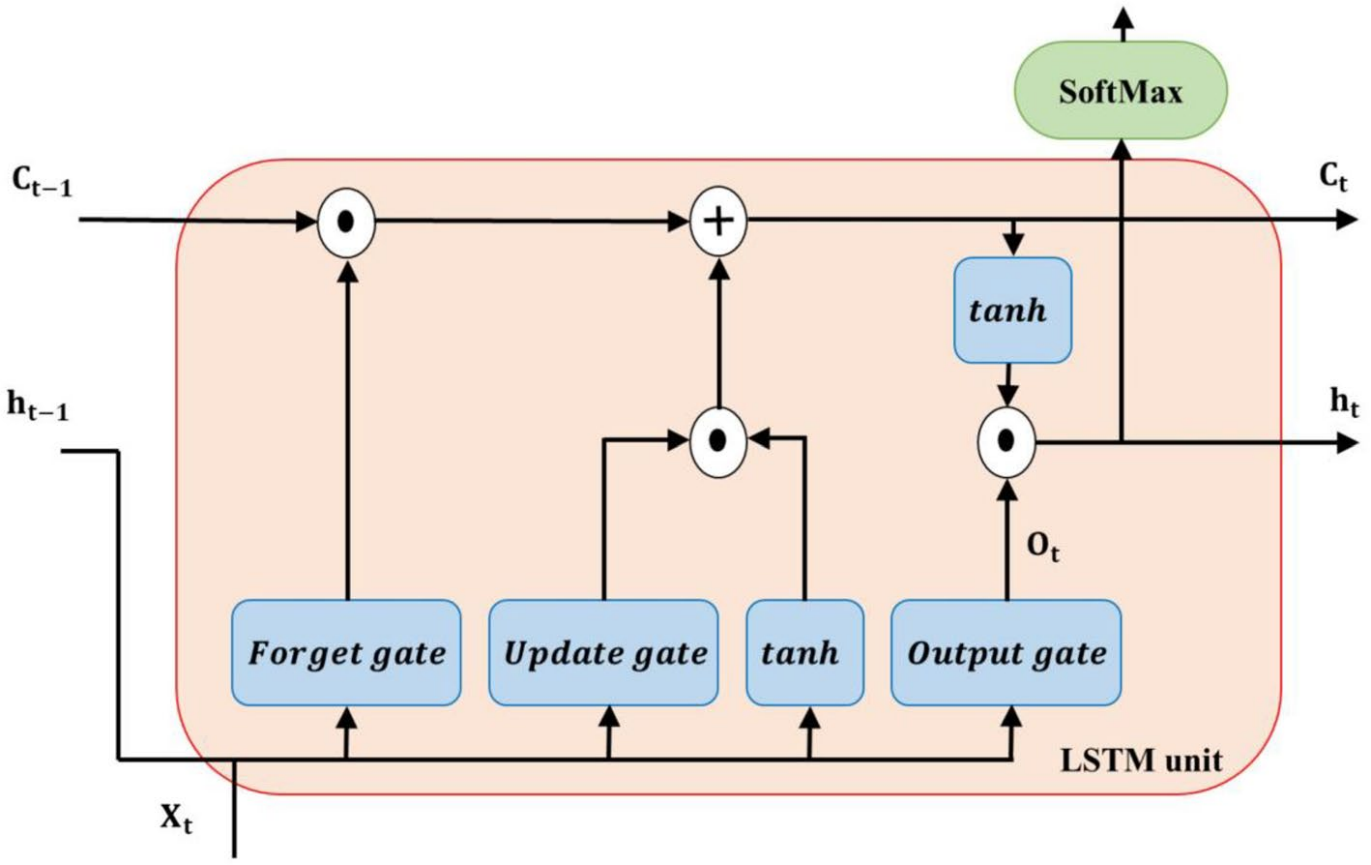
Architecture of LSTM unit.

A special kind of recurrent neural network that can capture dependencies over long time spans is a long short-term memory-based network (LSTM). The problem of long-term dependence was the reason for designing these networks. Remembering information for long periods is the default and normal behavior of these networks, and their internal structure is designed in such a way that they can learn information from far away, so this feature is hidden in their structure. LSTMs also have a sequence or chain-like structure, but the repeating module has a different structure. They have four neural network layers similar to RNN which these layers can be seen in Fig. 5. These layers interact and communicate with each other according to the special structure of these layers. This network introduces new concepts that were not present in the conventional recurrent neural network. Figure 6 illustrates the internal structure of this network. In these networks, there are so-called three gates (forget, update or input, and output gate) that control the data flow within the network^59^.

In addition, there is a memory cell, the network also has an input from the cache or ${h}_{t-1}$ and an input or $x$ and produces two outputs (one output is ${c}_{t}$ and the other output is ${h}_{t}$ which itself splits into two parts, one part goes to the following stage and another is used to generate output in the present stage if required). The forgetting gate regulates the information flow from the previous stage. This gate decides whether to use or not use the memory information from the former stage and, if something should be entered from the previous time step, how much should it be. The update gateway controls the flow of new information. This gate chooses to accept or reject new information in the present stage and if so, how much. This gate is generally called the input gate. The output gate also decides the amount of information from the former and present stage that will be passed to the following stage^60^. Therefore, LSTM works as follows^59^:

7 $${c}_{t}={f}_{t} {c}_{t-1}+{i}_{t} {\widehat{c}}_{t}$$

8 $${o}_{t}=\sigma \left({w}_{o} \left[{h}_{t-1},{x}_{t}\right]+{b}_{o}\right)$$

9 $${h}_{t}={o}_{t} \mathrm{tanh}({c}_{t})$$

10 $${y}_{t}=Softmax({o}_{t})$$

Where $\sigma$ represents the sigmoid function and ${o}_{t}$,${w}_{o}$, ${b}_{o},$ and ${y}_{t}$ are output, weight matrix, output gate’s bias at time t, and the possible output word, respectively. ${f}_{t}$ and ${i}_{t}$ are activation vectors of forget and input gates, and ${\widehat{c}}_{t}$ is the vector that feeds into the cell. These parameters are obtained from the following relations^60^:

11 $${f}_{t}=\sigma \left({w}_{f} \left[{h}_{t-1},{x}_{t}\right]+{b}_{f}\right)$$

12 $${i}_{t}=\sigma \left({w}_{i} \left[{h}_{t-1},{x}_{t}\right]+{b}_{i}\right)$$

13 $${\widehat{c}}_{t}=\mathrm{tanh}({w}_{c} \left[{h}_{t-1},{x}_{t}\right]+{b}_{c})$$

In more advanced and modified LSTM, the gate outputs (${f}_{t}, {i}_{t}, {o}_{t}$) are also the current cell state function (${h}_{t}$)^61^.

**Figure 7:**
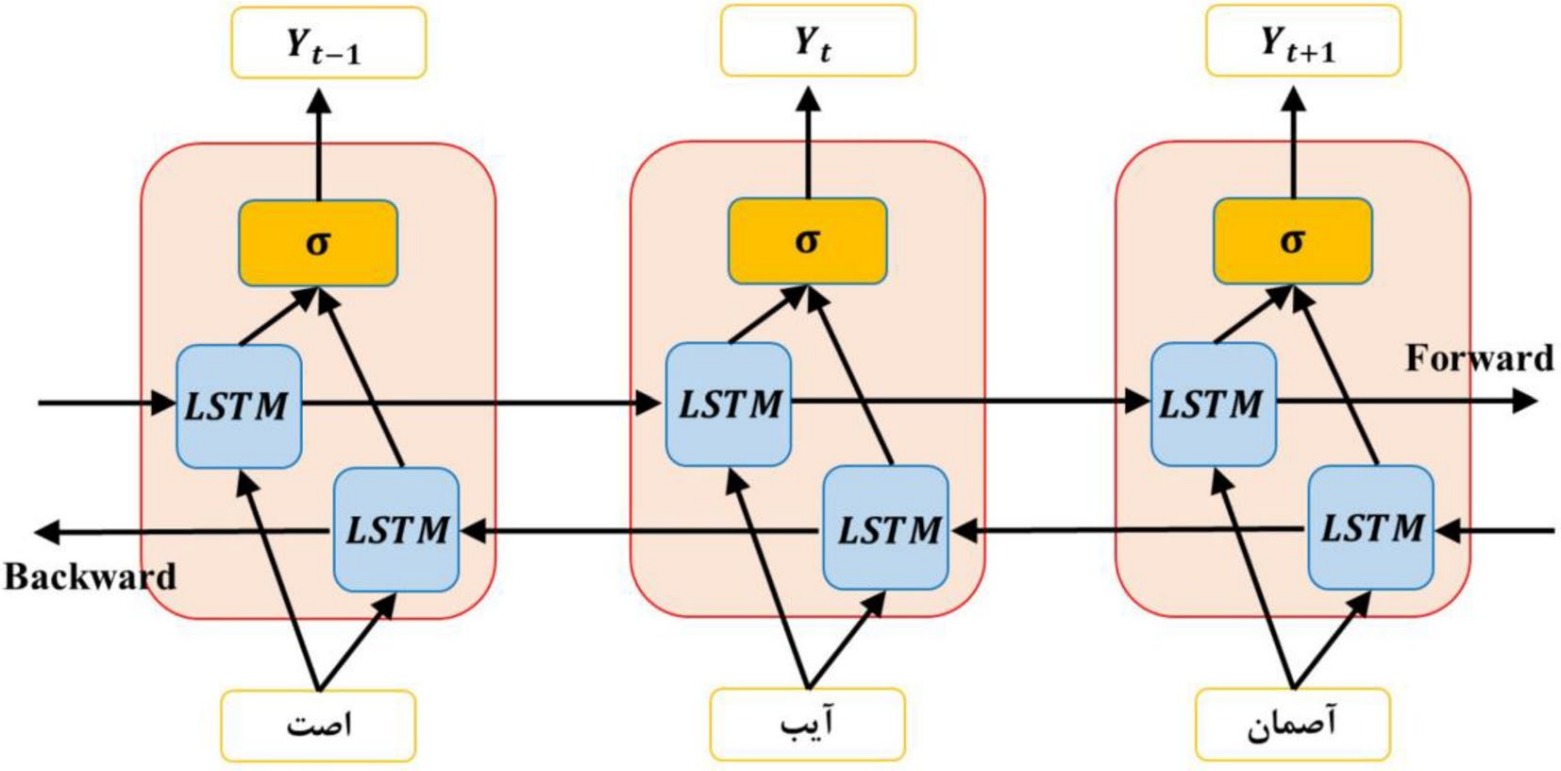
Bidirectional LSTM network architecture.

Bidirectional LSTMs are a more advanced model of this type of network, which, besides considering more distant dependencies, access information in both directions, enhancing their performance^62^. The bidirectional LSTM is an evolved type of LSTM with two separate recursive hidden layers. Each hidden layer has blocks of LSTM memory. There is no connection between these two hidden layers, and the two hidden layers are connected to one output layer. In this research, these types of networks have been used^63,64^. Figure 7 shows how connections are made in this type of network. Therefore, in these networks, the output is obtained from combining the forward and backward results, so^63^:

14 $${h}_{t}=\left[{h}_{t}^{f},{h}_{t}^{b}\right]$$

where^63^:

15 $${h}_{t}^{f}=LSTM\left({x}_{t}, {h}_{t-1}^{f}\right)$$

16 $${h}_{t}^{b}=LSTM\left({x}_{t}, {h}_{t-1}^{b}\right)$$

### Decoder

The decoder uses the intermediate data sequence generated in the encoder and produces a set of final outputs. The kinds of neural networks that can be useful for different tasks in decoding are described below^62,65,66^.

#### Neural network for decoding words without mistakes

In this part, a recurrent neural network, LSTM or bidirectional LSTM can be utilized. The advantages and disadvantages of them were presented in the previous section. This research employed a neural network that utilized Bidirectional LSTM. The number of memory units is very important in the learning power and recall of far information. So, its enhancement improves performance and increases the computing cost^62^.

#### Neural network for converting decoder output vectors into correctly spelled words

In the final part of the decoder, multilayer perceptron networks (MLPs) are usually implemented. An MLP consists of a minimum of three layers (input, output, and hidden layers). Each layer contains a group of neurons responsible for converting and transferring information from the preceding layer to the following layer. In the structure of this type of network, the neurons of a layer do not interact with other neurons in the same layer. This network uses nonlinear activation functions. All the neurons in a layer are linked to every neuron in the following layer, forming a fully connected network. They are the most basic kind of feed-forward neural network^65^.

### Deep learning

A sub-branch of machine learning, deep learning (DL) employs algorithms that aim to represent high-level abstract notions in the data. A deep graph with various processing layers that do linear and nonlinear transformations is used by this process. In other words, it learns knowledge and features in several layers. Deep learning is the term for researching new techniques for ANNs. It means using deep neural networks on huge data sets to learn a procedure to deal with a task. This method can vary from simple classification to complex reasoning. Multiple hidden layers are present in a neural network that is called a deep neural network (DNN). In a DNN, each layer works on a specific data feature. The deeper we go into the network, the more complex features the network will be able to recognize. This process is called feature hierarchy. This neural network feature makes them powerful and processes data with multiple features^66^.

### Evaluation measures

The performance of a text system is measured using different parameters such as accuracy, recall, precision, and F-criterion. Another parameter called Bleu number is also applied in machine translation. In this study, this parameter was also utilized to check the performance of spelling correction due to the similarity and closeness of spelling correction to machine translation. These evaluation parameters show how well the performance of the developed model is appropriate in textual data analysis. There are four different modes for the model to respond to the actual model. Since the model aims to identify and correct misspelled words, we consider misspelled word identification as positive like Singh and Singh study^46^. In our application, these states are: the real phrase has a misspelling and the network or rules have edited it correctly (TP), the real phrase has a misspelling but the network or the rules have not edited it correctly or it has not recognized incorrectly (FP), the real phrase is without spelling mistakes and the network or rules correctly recognized it without mistakes (TN), and the real phrase is without spelling mistakes but the network recognized it as having a spelling mistake (FN). In the following, various criteria for evaluating the performance of text systems and their calculation methods are described based on these states^46,50,68^.

The accuracy criterion expresses the ratio of inputs that the process correctly recognized out of all the predictions that the same process made. In other words, it means^46^:

17 $$\mathrm{Accuracy\%}=\frac{TP+TN}{TP+TN+FP+FN}\%$$

The recall criterion expresses the proportion of detections that were picked by the model among the total correct detection. Therefore, it is calculated from the following relationship^50^:

18 $$\mathrm{Recall\%}=\frac{TP}{TP+FN}\%$$

The precision criterion evaluates the proportion of words that had the right classification among the total positive detections forecasted. Therefore, this criterion is calculated through the following relationship^50^:

19 $$\mathrm{Precision\%}=\frac{TP}{TP+FP}\%$$

The F-measure criterion merges precision and recall factors and evaluates how good a model is in both precision and recall metrics. The harmonic measure of precision and recall elements is another name for this criterion. Calculation of this parameter in terms of two other parameters is as follows^46^:

20 $$\text{F-Measure\%}=2\frac{Precision . \mathrm{Recall}}{Precision+Recall}\mathrm{\%}$$

Bilingual evaluation understudy criteria (Bleu Criteria) is a method for evaluating the quality of the machine-translated text. In this criterion, in order to compare the machine translation with the original translation, the modified form of precisions is used. For this purpose, the overlap of n-grams of machine output text and reference text is identified and the precisions value of n-grams with different gram sizes from 1 to 4 is calculated. Using these precisions, the Bleu value is calculated from the equation^68^:

21 $$BLEU=\mathrm{min}\left(1,\frac{output - length}{reference - length}\right)\times {\left(\prod_{i=1}^{4}{precision}_{i}\right)}^{0.25}$$

A number ranging from 0 to 1 is the output of Bleu, which reveals the similarity between the candidate text and the reference texts. The closer the number is to one, the greater the similarity.

## Materials and method

**Figure 8:**
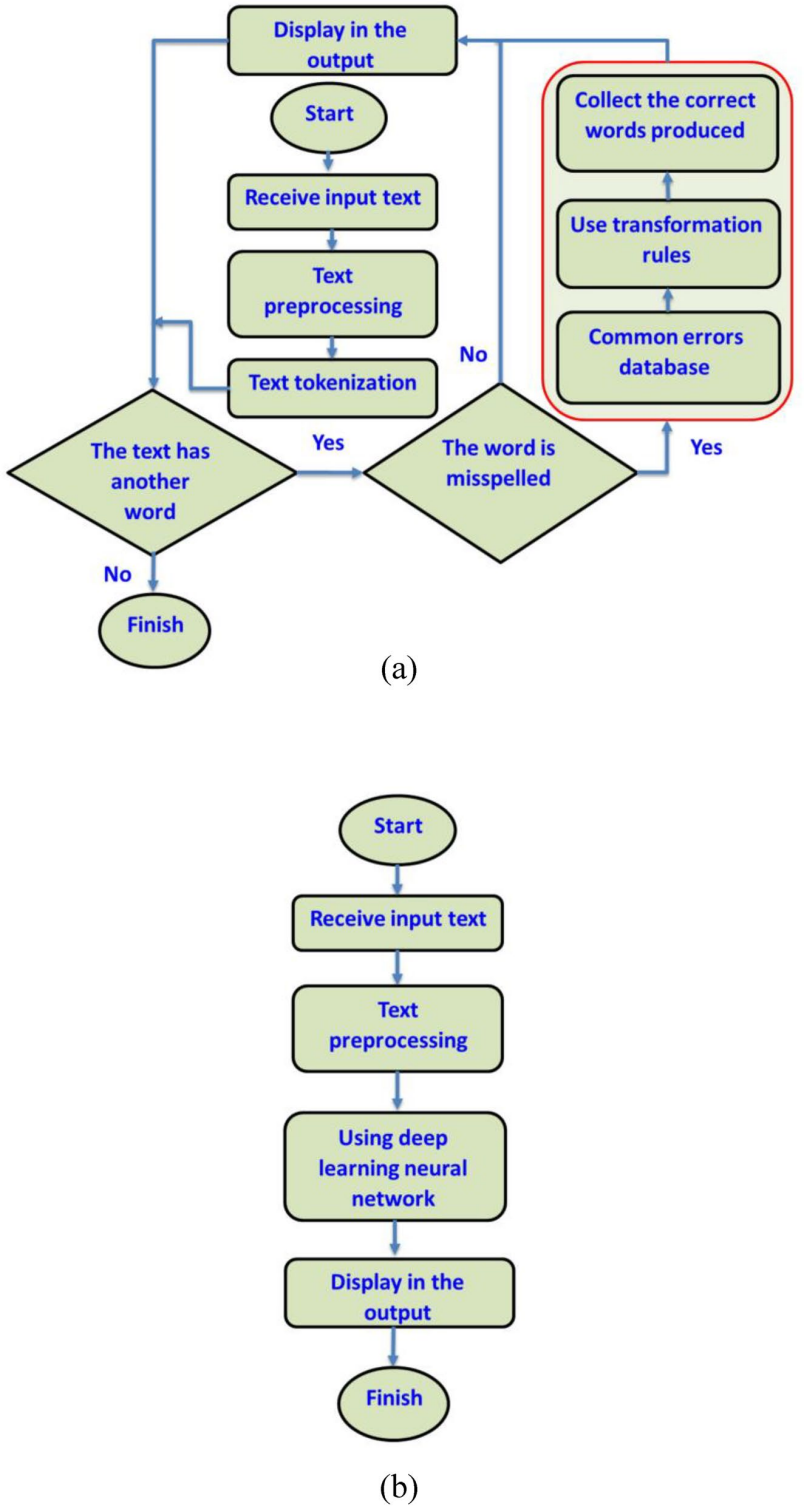
Spelling correction flowchart of (**a**) rule-based, (**b**) deep learning.

Figure 8 illustrates the flowchart of error correction with two methods: rule-based and deep learning. The system receives sentences from the input and performs preprocessing and tokenization on the text. Then, it detects and corrects the error and displays the result in the output. This research proposes a system for correcting spelling errors that consists of three main components: input data preprocessing, error detection, and error correction. Each language has its own preprocessing requirements. The hazm library^69^ was used for data preprocessing. In the rule-based method, the system searches for words with misspellings in the database. If a word is not found in the database, it is misspelled and the system corrects it based on the rules. Before that, the system fixes common spelling errors using a list. The system trains a neural network using an artificial database in the deep learning method. The process of creating a synthetic database is explained in "Artificial database construction for learning and deep networks structures" section. The system has a user-friendly UI based on Django, which allows users to enter text and view the output text generated by the system.

### Preprocessing and database of Persian words

As mentioned, the hazm library was used for preprocessing. The hazm is a standard library for preprocessing in the Persian language. The three main digestion modules that were used in this research are Stemmer (extract the root of the noun), Lemmatizer (extract the root of the verb), and Normalization (transform a text into a standard form). For Stemmer, a list is used that specifies the prefix and suffix that can be attached to the nouns, and if one of them appears in the words, it is removed. The Lemmatizer is more complex than Stemmer, and besides the unit of tokenization and root finder, several auxiliary verbs such as is, was and other verbs were used. The current suffixes and prefixes are stored in the form of a list and separate auxiliary verbs from the main verb. In the normalization unit, the extra space is removed by suffixes and prefixes. In fact, in Persian, the prefix and suffix of the words include a half space, but a space is mistakenly placed instead, so this extra space should be removed and by identifying this prefix and suffix, this space can be removed.

The database of Persian words used is the database of Zaya words of the Persian language^70^, a standard library with common and usual words. The vocabulary in this database includes about 55 thousand entries. In each entry, information related to the word's written form in the Persian script, phonetic construction, lexical category, stress pattern and frequency of the word is stored. A text corpus containing 10 million words was used to prepare this database. This corpus included 100 thousand words with different frequencies, and by removing inflectional forms, the number of these words was reduced to about 44 thousand words. Due to the lack of some common words and completely scientific words in this list, Zaya words were compared with Persian culture and about 11 thousand new entries were added to it. And finally, the Zaya database with 55 thousand distinct words was obtained^67^.

### List of common misspellings

About 700 incorrect words were collected that were used as common misspelled words. This list is derived from several sources, the main one of which is the Wikipedia site, the error finder section. All the words detected as wrong should be checked with these words; if they exist in this list, they should be changed into their correct ones. Some of these words and their common mistake forms are displayed in Table 1.

**Table 1:**
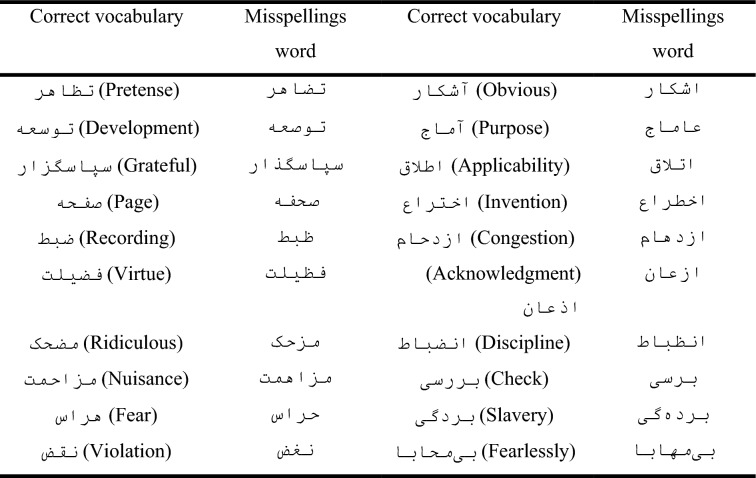
Examples of words in the common misspelling list.

### Rules for text correction

In general, we have two types of errors in texts, one is phonic and the other is typographical. Phonic mistakes are limited and occur in the wrong spelling of homophonic letters in the Persian language. To implement it, we tried to consider all vowel letters, which was implemented by replacing each vowel letter with a letter that has the same sound as it. It should be noted that only one vowel letter is considered in each rule.

There are many types of typos, which can be classified according to Table 2 based on the research of Shahmiri et al.^71^. We tried to take into account all these typographical errors, and implement them. In Table 2, the additional distance means inserting a space between the two main letters of the word and removing the distance means removing the space between two words and becoming one mistake word. In the following, the implementation of each of the errors is discussed.

**Table 2:**
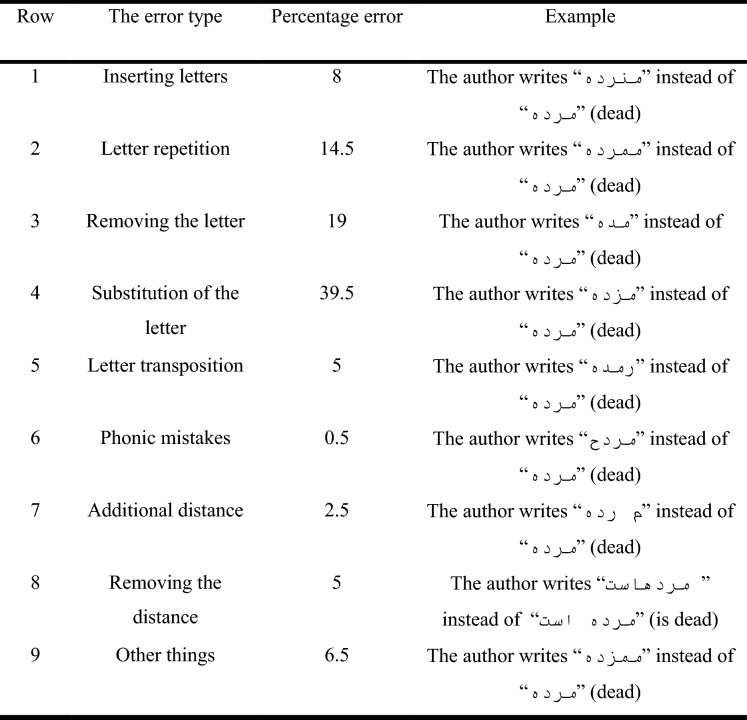
Types of mistakes and their occurrence ratio in the Persian language^71^.

#### Substitution of the letter

Instead of one of the main letters, another key was pressed on the keyboard. The pressed key is often close to the main key. Therefore, to create this error, the right and left keys were considered for each letter and it was assumed that the right or left key would be pressed instead of that key.

#### Letter transposition

One of the main letters should be replaced with the next or previous letter in the word itself. For this, each sentence in the database is broken into words and in each word, every time the letters of the word are read from the database, the first letter is replaced with the second, then the second with the third, and in this way. If there are sufficient letters for the word, the first five will be considered for this process. That is, 5 different misspellings were generated for each word.

#### Letter repetition

One of the main letters should be written twice. To create this error, each sentence was converted into words, then one of the letters of the word was repeated every time the text was read, for example, the first letter of each word was repeated in the first turn. In the next reading, the second letter of the word was repeated and so on until the fifth letter.

#### Removing the letter

One of the main letters of the word should not be typed, its implementation method is similar to repeating a letter, only here, instead of repeating a letter, that letter is deleted every time the text is read. That means the first letter of each word is deleted in the first round, and so on. It is continued until the fifth letter.

#### Additional distance

Insert a space between the two main letters of the word. It has been done in the same way as before, that every time reading the text, a space was considered between two consecutive letters of the word, for example, in the first reading, a space was inserted between letters one and two, and so on.

#### Inserting letters

It happens when a letter is written between two main letters during the writing and this letter will be close to the main letters. In order to implement this error, it was done according to the repetition of the letter, only instead of repeating the main letter, based on what the letter was, the letter of the right or left button on the keyboard was pressed when typing.

In this research, we tried to cover all the errors, therefore, by implementing the rules related to these errors, 112 incorrect sentences of different types of errors were created for each correct sentence. In this way, the necessary corpus for neural network training was obtained. Part of the study is error correction with the help of rules, the same rules were used for spelling correction, for example, if a letter has been removed as an error in a word, we must add a letter in the correction section and other rules can also be utilized similar to this. Several examples of error sentences corrected by the rules and database of common errors are indicated in Table 3.

**Table 3:**
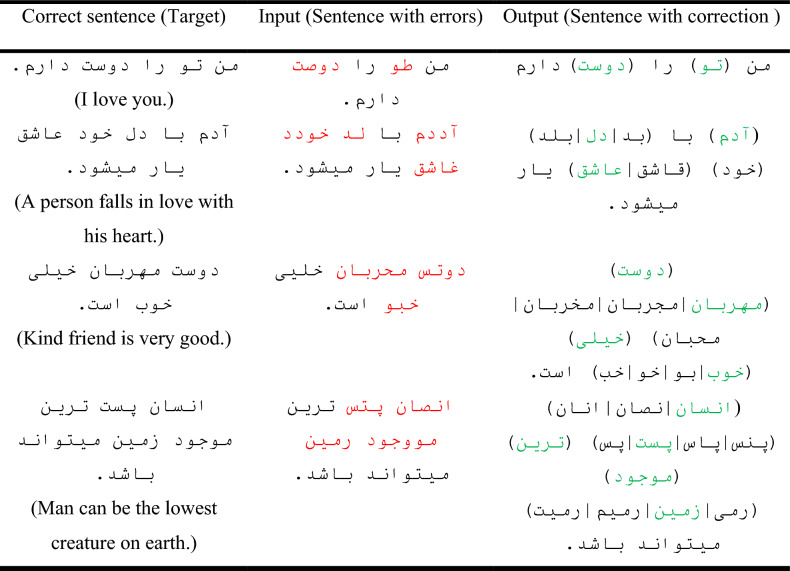
Examples of sentence correction by rules.

### Artificial database construction for learning and deep networks structures

Using a corruptor is one way to create a training database for the correction of misspelled. Alian et al.^72^ used a corruptor to error generation in words for the Arabic language. All the spelling mistakes that are considered in the previous section were tried to be made artificially by rules. A database of 10 thousand sentences without spelling mistakes, which was the sum of the official sentences of the standard database of the Persian to English translation^73^ and the database of official sentences obtained from the subtitle collection of different movies, was considered. Then according to the conducted studies, 112 rules were applied to each sentence that each rule produce errors in a different way. By doing this, a database with about 1 million and 200 thousand sentences with misspellings was created. One million sentences of this database were applied in the neural network section for training and testing.

The deep learning method was used to map the sequence to the sequence (transforming incorrect text into correct text). The word embedding method based on FastText^74^ was also applied in the neural network. FastText is a library for learning word embedding and text classification, which was developed by Facebook’s artificial intelligence laboratory and uses neural networks for word embedding. In this study, the trained Persian language model that was provided by the Facebook artificial intelligence laboratory team was used.

The artificial database that was created was used to train and test the final DNN model. The training and testing data consisted of 800 thousand and 200 thousand sentences, respectively. The parameters that were set in the ANN are shown in Table 4. The default embedding layer was turned off and FastText was activated as the embedding layer. The model was trained by the Adam algorithm. This method can deal with the gradient reduction problem in noisy data. This algorithm is very popular in deep learning because of its fast learning ability. The value that a model should aim to reduce during training is computed by the loss function. The sparse categorical cross entropy was chosen as the loss function, which is defined by the following equation^75^:

23 $$Loss=\frac{-1}{N}\sum_{s\in S}\sum_{c\in C}{l}_{s\in c}\mathrm{logp}(s\in c)$$

where S are samples and C are classes and each sample belongs to a class. ${l}_{s\in c}$ is the function that indicates whether sample s is in class c or not. $\mathrm{p}(s\in c)$ is the probability that the model gives for sample s being in class c.

**Table 4:** Selected characteristics for network training.

Criterion	Value/function
Embedding	FastText
Optimizer	Adam
Loss	sparse_categorical_crossentropy
Metrics	acc
Epochs	100
Batch_Size	8
EarlyStopping	3
Monitor	val_loss

After several times of trials and errors, the batch size was selected to 10. To stop training, two conditions were considered: 100 training epochs or failure to improve in three consecutive epochs. In all cases, the second condition was established.

**Figure 9:**
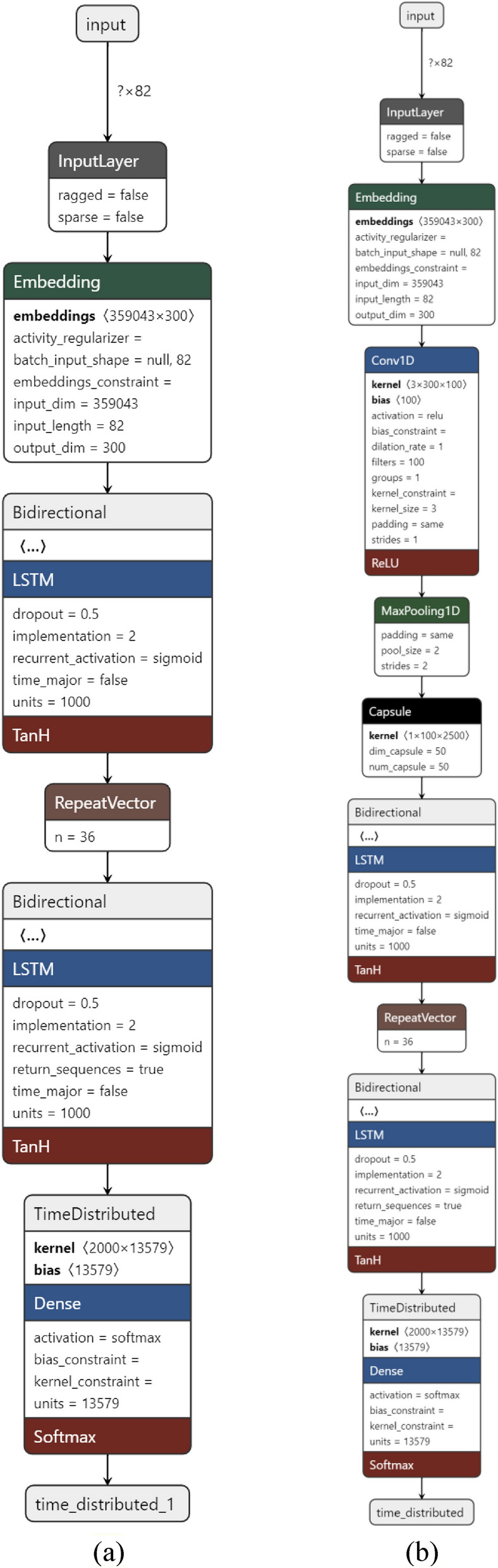
The structure of the neural networks employed in this study (a) basic network (LSTM unit) (b) basic network (LSTM unit) with capsule and convolution layer.

The architecture of the implemented network types in this research is presented in Fig. 9. The basic encoder-decoder architecture is exhibited in Fig. 9a, and Fig. 9b shows the types of layers added to improve performance. In Fig. 9a, first, all the sentences are converted into integers, then they are given to the encoder-decoder-based network to map this sequence (the text with errors) to the target sequence (corrected text). At the input of this network, a FastText-based word embedding layer was placed, which converted integers into vectors with dimensions of 300 dimensions. After using the word embedding layer in the encoder section, Bidirectional LSTM units have been applied. A repeating vector layer was employed to transform the encoder output into the decoder input. In fact, with this vector, it is possible to separate different sequences for the decoder. Then the decoding section is started where there is a layer with bidirectional LSTM. After that, a time distribution layer was placed to extract the sequences and map vectors to words. In Fig. 9b, two layers of convolutional and max pooling are utilized to apply various filters, remove noise, and letter repetition errors, and extract the main feature. The capsule layer was employed to maintain relationships between words. After the word embedding layer, these layers were appended to the encoder section, and then a layer of LSTM units followed them.

### User interface

**Figure 10:**
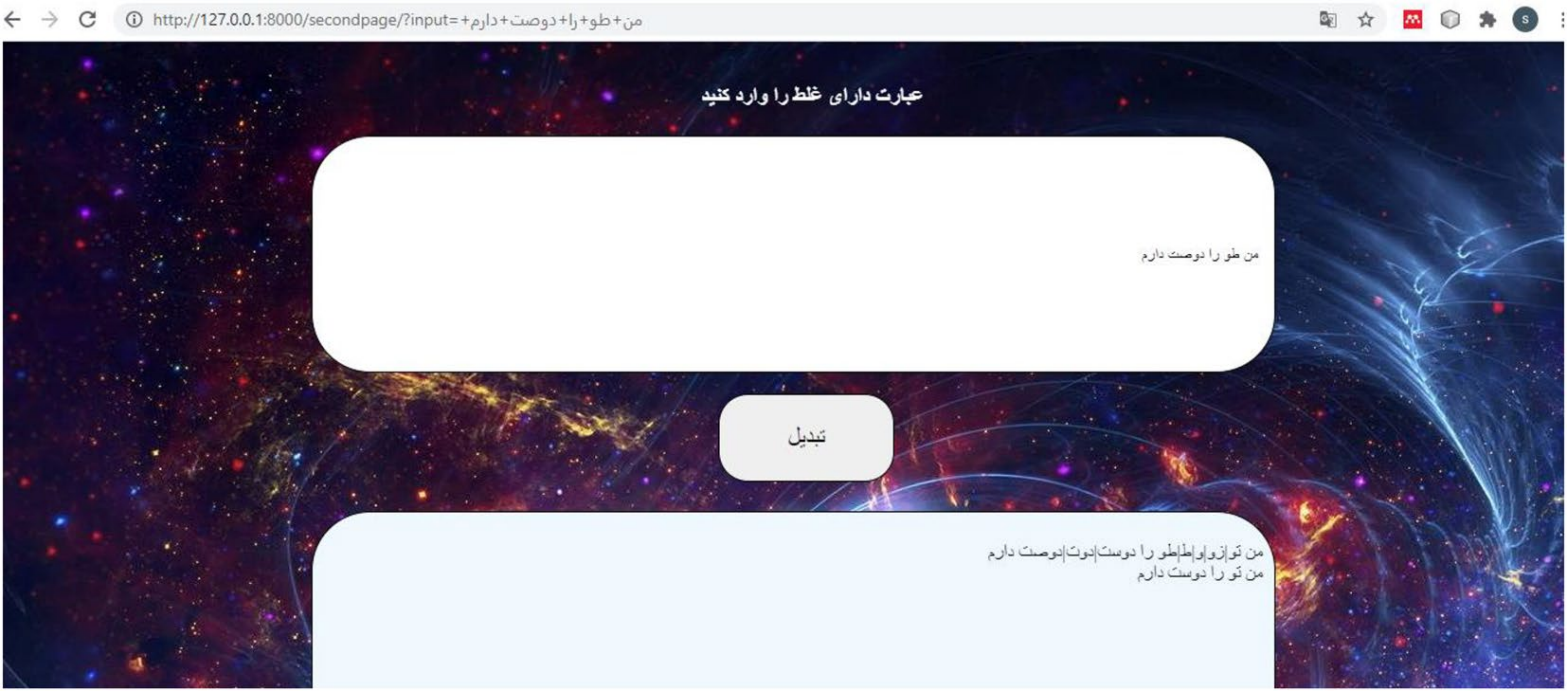
The user interface implemented in this study.

In practice, the system requires the input of sentences with misspellings by the user and the output of corrected sentences. A web-based user interface was implemented by the Django framework of python language. Django^22^ is a free and open-source web framework that uses Python and follows the model-view-controller pattern. Django aims to make it easy to build complex and database-driven websites, and it is designed based on the reusability and connect ability of different components, rapid development, and doesn't repeat yourself. The “doesn't repeat yourself” principle in software development focuses on minimizing repetition of information. It achieves this by replacing redundant details with more stable abstractions or by utilizing data normalization to prevent redundancy. Django uses Python throughout, even for configuration, files, and data models. The designed user interface is shown in Fig. 10. This user interface is actually a web page that is connected to our server. Input and output are received and sent by the page on the front-end side, and the necessary processing is performed on the server side, which is the back-end side. The user enters his sentences in the specified box and sees them corrected in the bottom box. The output contains two texts, one text is related to correction using rules and the other is related to modification by deep learning.

## Result and discussion

**Figure 11:**
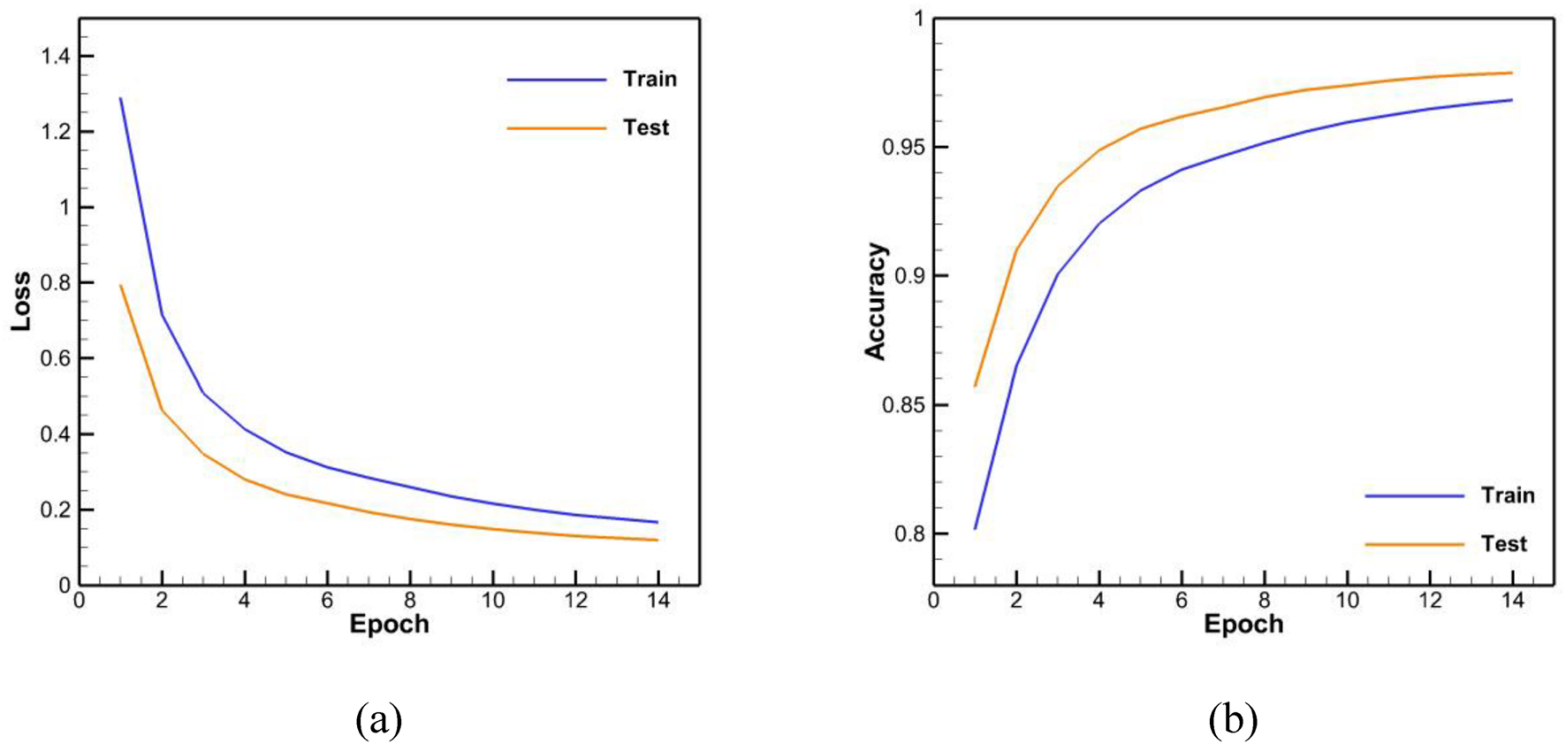
Base network with 500 units of memory per layer (**a**) loss function, (**b**) accuracy.

**Figure 12:**
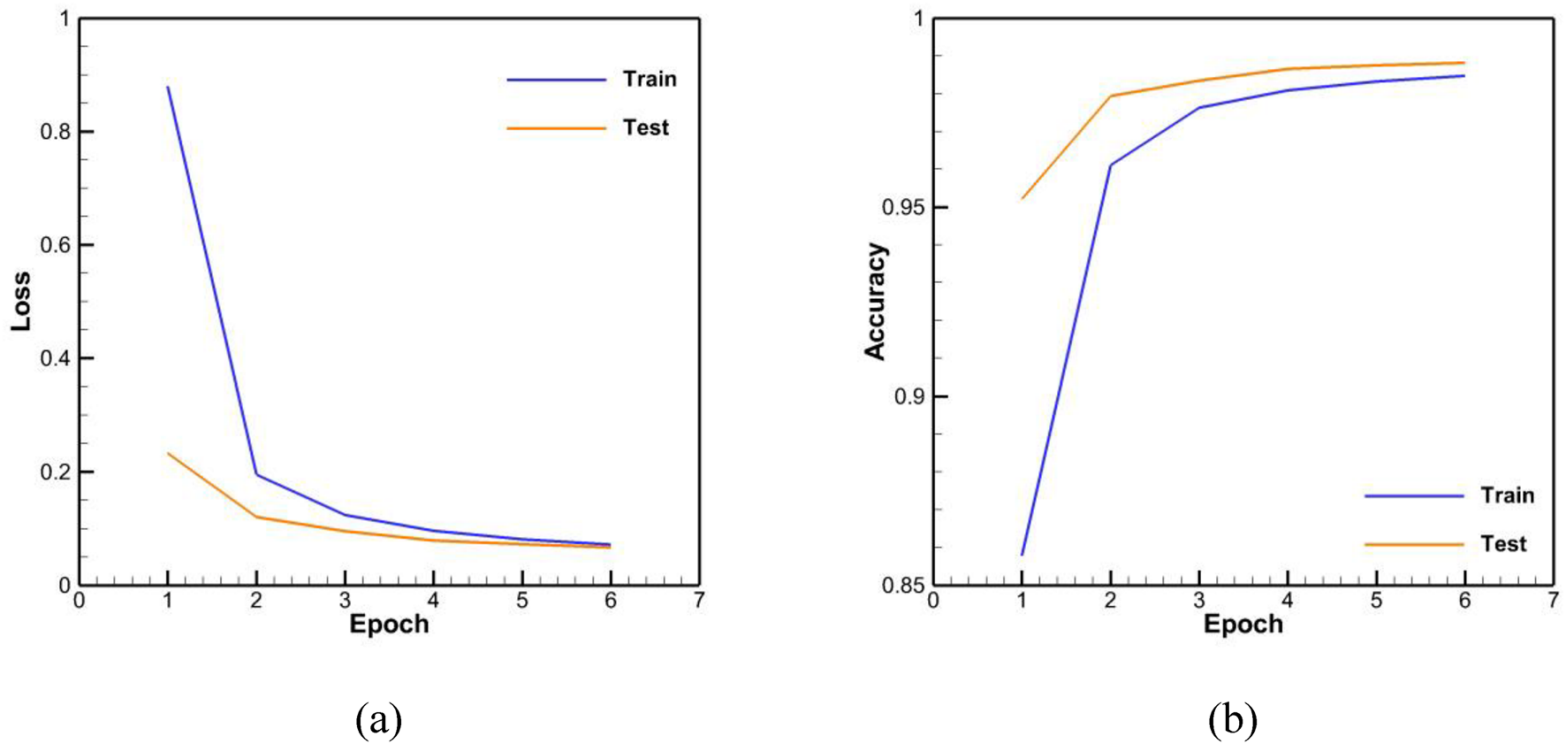
Base network with 1000 units of memory per layer (**a**) loss function, (**b**) accuracy.

**Figure 13:**
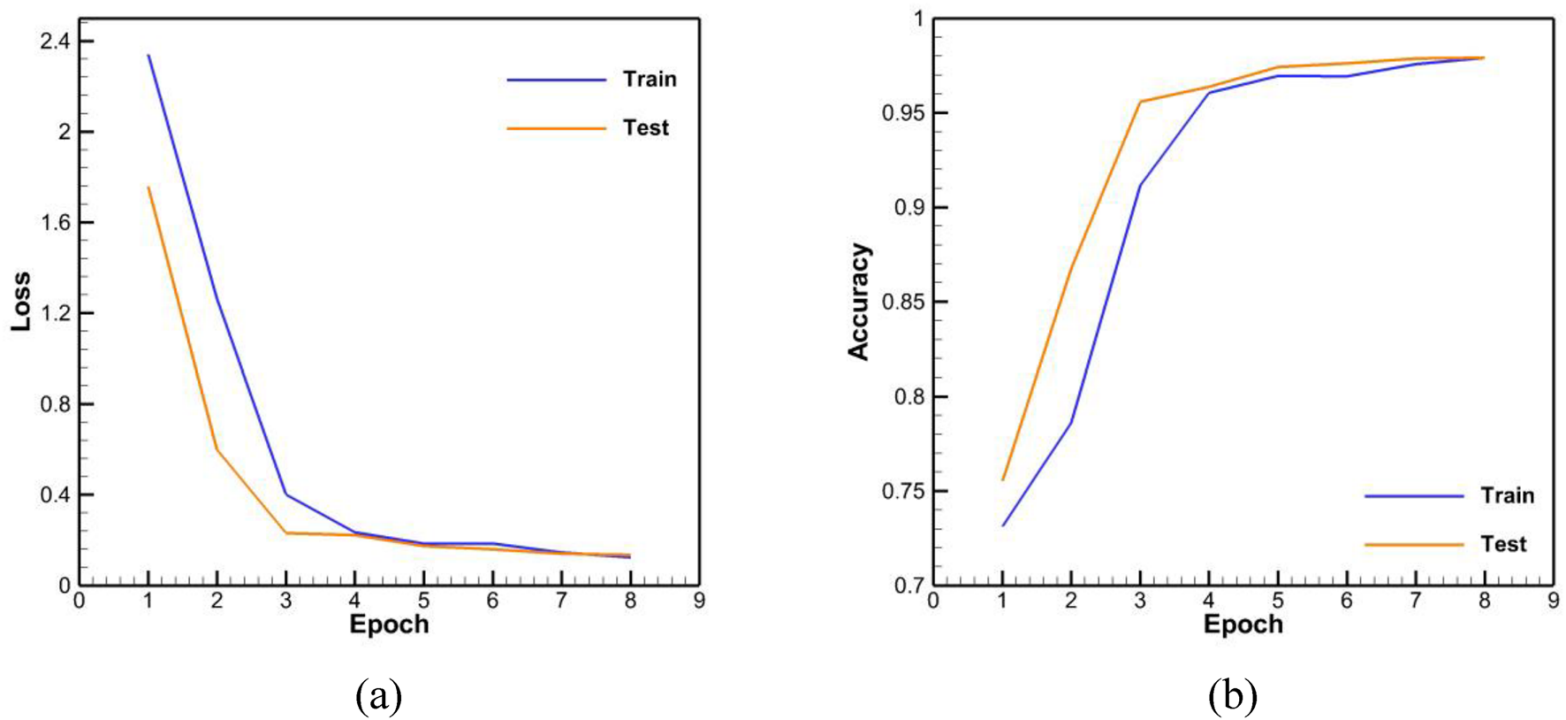
Network with 1000 units of memory per layer with capsule and convolutional layers (**a**) loss function, (**b**) accuracy.

The accuracy results of the trained networks are presented in Table 5. The loss and accuracy diagrams related to these networks are also shown in Figs. 11, 12, 13. It should be noted that an epoch includes a complete training cycle in the training set, in other words, when every sample of the training set has been viewed, an epoch is finished. As can be seen in Figs. 11, 12, 13 and Table 5, in general, the three networks have acted almost identically. According to Fig. 12 and Table 5, by increasing the number of LSTM blocks of the encoder and decoder layers, the performance has improved and it has been able to achieve more accuracy than the previous state with the half number of epochs, which is both in the accuracy of the test and training data and in their loss function is observed. In the diagram of loss and accuracy of Fig. 12, we see graphs were closer to each other in this case compared to the case with less LSTM block number. This subject indicates an improvement in the speed of training, and according to the figure, the number of epochs for training has decreased to 6 for this case. On the other hand, according to Table 5, the accuracy of training and testing for 1000 LSTM blocks is higher than 500 LSTM blocks.

**Table 5:** Accuracy of training and testing data in trained networks.

Network type	Training accuracy	Testing accuracy
Basic neural network with 500 memory units per layer	0.968	0.978
Basic neural network with 1000 memory units per layer	0.984	0.988
Neural network with 1000 memory units per layer along with encapsulation and convolution layer	0.979	0.979

According to Fig. 13 and Table 5, the performance of the network with capsular and convolutional layers is similar to the basic network based on LSTM. This closeness is due to the fact bidirectional LSTM units have the ability to preserve the order of their previous and next words, and to some extent, they can also perform the task of the capsule network. On the other hand, LSTM units can extract features to some extent by memorizing long strings. The basic network with 1000 LSTM units in each encoder and decoder part was considered as the final network topology. Examples of the output of this network in spelling correction are reported in Table 6. According to this, all kinds of errors have been in these sentences. The network has been able to correctly identify these errors and replace the correct phrase due to the high accuracy of the learning.

**Table 6:**
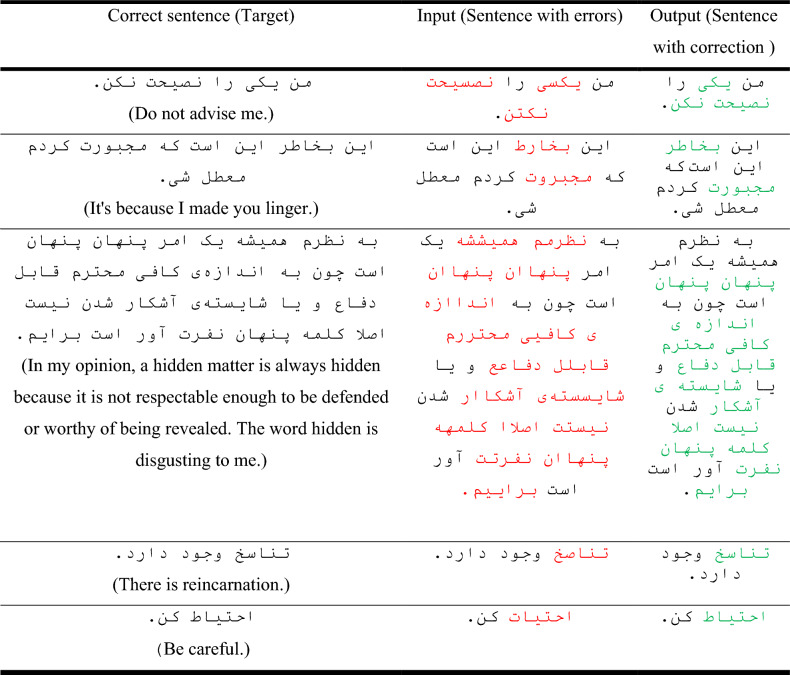
Examples of sentences correction by the trained network.

In Singh and Singh^46^, which has done the spelling correction for the Indian language, a part of the corpus was considered as evaluation data. They obtained and reported various evaluation criteria such as accuracy, precision, recall, and F-criterion. We also evaluated the system by calculating all these parameters, and the Bleu criterion. 200 thousand data were used to evaluate the approach based on deep networks. But since it was not possible to apply this number of sentences to the other approaches, we compared and checked the evaluations with 2500 sentences. In the rule-based approach and the list of common errors, as different rules suggest different words for a wrong word, therefore Levenshtein's distance algorithm was used. The suggested words were sorted based on that and the word with the highest ranking was chosen for evaluation. As we know, to transform one string to another string, the least number of operations that are required is the Levenshtein distance between two strings, and an operation can be either inserting or substituting a character, or switching two characters. Therefore, it is a good criterion for choosing one or more words from among several suggested words in error correction. Table 7 displays the results of the evaluation for spelling correction. The table shows that the network’s basic architecture had the best performance. The basic network with 200 thousand evaluation data was able to correct 70% of the errors and increase the accuracy of the text to 87%. The weaker performance of the rules is due to the initial database, which does not cover all the words. Arab Surkhi et al.^62^ and Yazdani et al.^76^ have pointed out the impact of the database on the evaluation result in the rule-based method. In addition, only the lowest Levenshtein distance has been utilized for evaluation, while a suggested list is presented to the user. Hu et al.^41^ indicated that when using the suggested list, the actual system performance will become higher than the lowest Levenshtein distance suggested. As one can observe, the performance of the network with capsular and convolutional layers is similar to the basic network, and in the evaluation, both have performed almost the same.

**Table 7:** Evaluation of different methods used in this research.

Type of approach	Accuracy (%)	Precision (%)	Recall (%)	F-Measure (%)	Bleu (%)
Rules (with 2500 sentences)	62	21	33	25	40
Basic network (with 2500 sentences)	64	50	48	49	65
Network with convolutional layer and capsule (with 2500 sentences)	60	43	40	41	60
Basic network (with 200 thousand sentences)	87	70	89	78	84

To show the adequacy and goodness of the network's performance, the operation of the neural network-based system was compared with similar systems (spelling correction) for other languages. The purpose of this comparison is to validate and ensure the sufficiently of the network activity in the evaluation, the result of which can be seen in Table 8. In this comparison, Singh and Singh study^46^ was used, which contrasted its system with similar systems and reported the results. As shown in the table below, the network-based approach is consistent with the results of similar studies for other languages, and the proposed model gives better performance than other current models for spell-checking and correction.

**Table 8:** Comparison of network-based system performance of this research with similar systems expressed in Singh and Singh's study^46^.

Method type	Accuracy (%)	Precision (%)	Recall (%)	F-Measure (%)	Bleu (%)
LSTM model (Malayalam)	55	47	97	63	–
HINSPELL (Hindi)	77	–	–	–	–
CNN-Strides (English)	48	46	40	42	–
CNN-Filters (English)	55	–	–	–	–
HINDIA (split dataset)	80	85	72	78	–
HINDIA (testing dataset)	74	81	72	77	–
Our network (Persian dataset)	87	70	89	78	84

## Summary and conclusion

Due to the lack of a comprehensive system for the automatic correction of Persian texts and considering the much study to create such systems in the English language, the creation of an automatic system for spelling correction of Persian texts was investigated in this study. In order to correct the error, the error must first be identified. Therefore, a database that includes the main and more useful Persian words was collected. After the initial pre-processing of the input data, the words were compared with the database. If the word is not found in the database, it is recognized as an error. In the proposed system of this study, error correction is done utilizing three approaches. In this research, a list of commonly misspelled words including 700 words was prepared so that if they are identified, they can be inserted and replaced directly. A database was compiled to detect spelling errors based on rules, and this database included 55 thousand common Persian words. 112 rules were implemented to correct spelling caused by phonetic similarity and typographical errors, which provided suggested words for unrecognized words in the database. In order to evaluate, 2500 spell error sentences were applied and the word with the lowest Levenshtein distance was chosen as the selected word in this method. In the deep learning approach, an encoder-decoder network based on LSTM units was employed along with word embedding in the input and convolutional, max pooling, and capsule layers. An artificial database containing about 1 million and 200 thousand data was constructed to train the deep neural network. 800 thousand sentences were considered for training, 200 thousand sentences were applied for testing, and the others were utilized for evaluation. The basic structure was considered an encoder-decoder network based on LSTM. The results indicated that the effectiveness of the network with capsular and CNN layers is similar to the basic network. The trained network performed well in the evaluation and obtained values of 87%, 70%, 89%, 78%, and 84 for accuracy, precision, recall, F-criterion, and Bleu's criterion, respectively. The results indicated that in spelling correction, the approach based on the deep learning is better than rules (The codes written in conducting this research are available inSupplementary Information).

### Supplementary Information

Supplementary Information.

## Data Availability

Data will be made available on request. If someone wants to request the data from this study, they can contact corresponding author.
