# Supplementary material for: Correcting spelling mistakes in Persian texts with rules and deep learning methods
Source: Sci Rep. 2023 Nov 15;13:19945. doi: 10.1038/s41598-023-47295-2 (PMC10652024; doi:10.1038/s41598-023-47295-2)
Supplement: Supplementary file 1 — Supplementary Information. [file 41598_2023_47295_MOESM1_ESM.pdf]

Attachments

Table A.1. html, css code of the designed User interface

```
<?xml version="1.0" encoding="UTF-8"?>
<!DOCTYPE html
        PUBLIC "-//W3C//DTD XHTML 1.0 Transitional//FA"
        "http://www.w3.org/TR/xhtml1/DTD/xhtml1-transitional.dtd">
{% load static %}
<html xmlns="http://www.w3.org/1999/xhtml" xml:lang="fa" lang="fa" dir="rtl" >
<head>

    <title>املائی غلط تبدیل</title>

    <style type="text/css">

       body{
           background: url("/static/images/3.jpg");
           width: 1920px;
           height: 1200px;

          }
         #t1{
              border-radius: 10px;
              border:1px solid black;
              padding-right: 10px;
              padding-left: 10px;

          }
         #t1{
             padding-bottom: 25px;
             padding-top: 25px;
             border-radius: 50px;
              position: fixed;
             top: 80px;
             right: 250px;
             width: 800px;
             height: 150px;

         }
         #tt{
             color: white;
             font-size: larger;
             position: fixed;
             top: 20px;
             right: 550px;

         }
        #cli{
             border:1px solid black;
              padding-right: 10px;
              padding-left: 10px;
              padding-bottom: 25px;
             padding-top: 25px;
             border-radius: 25px;
             position: fixed;
             top: 300px;
             right: 575px;
            width: 150px;
            font-size:20px ;

        }
        #div1{
              border:1px solid black;
```

```
              padding-right: 10px;
              padding-left: 10px;
              padding-bottom: 25px;
             padding-top: 25px;
             border-radius: 50px;
              position: fixed;
             top: 400px;
             right: 250px;
             width: 800px;
             height: 150px;
            background-color: aliceblue;
        }


    </style>
</head>
<body id="bood"  >

 <form action="http://127.0.0.1:8000/secondpage/" method="get" id="fo1">
  <h2 id="tt" lang="fa">عبارت دارای غلط را وارد کنید</h2>
   <input type="text" name="input"  id="t1" value="{{current_data}} "/>

         <input type="submit" value="تبدیل" id="cli"/>

 </form>
<div id="div1"> <div> {{process_data}} </div> <div> {{process_data1}} </div>
</div>
</body>
</html>
```

Table A.2. Code of the Django server section to receive and display the results

```
def secondpage(request):
    if 'input' in request.GET and request.GET['input']:
        now = request.GET['input']  # datetime.datetime.now()
        # from 1 to len()-2
        a = now.split(' ')
        # Simple way of using templates from the filesystem.
        # This is BAD because it doesn't account for missing files!
        loc =
(r"C:\Users\saman\PycharmProjects\untitled\aslidatasiroos\WordsSpellCheck.xlsx
")
        # To open Workbook
        wb = xlrd.open_workbook(loc)
        sheet = wb.sheet_by_index(0)
        word1 = []
        for word in a:
            jj = 0
            for i in range(sheet.nrows):
                if (str(sheet.cell_value(i, 0)) == str(word)):
                    jj = 1
                    word1.append(str(sheet.cell_value(i, 1)))
                    break
            if (jj == 0):
                word1.append(word)
        joint_word1 = ' '.join(word1)
        word2 = rule_checkspell(joint_word1.split(" "))
        sentencAnn=ann_siroos(now)
        fp = open('myproject\\second.xhtml', encoding='utf-8')
        t = Template(fp.read())
        fp.close()

        html = t.render(Context({'current_data': now, 'process_data':
word2,'process_data1':sentencAnn[0]}))
        return HttpResponse(html)
    else:
        # Simple way of using templates from the filesystem.
        # This is BAD because it doesn't account for missing files!
        fp = open('myproject\\second.xhtml', encoding='utf-8')
        t = Template(fp.read())
        fp.close()
        html = t.render(Context({'process_data': ''}))
        return HttpResponse(html)
```

Table A-3. Code of written rules for correcting and creating errors

1-

```
        def rule_checkspell(words):
    loc =
(r"C:\Users\saman\PycharmProjects\untitled\alsidaatasaman\database.xlsx")
    # To open Workbook
    wb = xlrd.open_workbook(loc)
    sheet = wb.sheet_by_index(0)
    sli = []
    normalizer = InformalNormalizer()
    lemmatizer = Lemmatizer()
    stemmer = Stemmer()
    set1 = []
    for i in range(sheet.nrows):
        set1.append(sheet.cell_value(i, 0))
    # set1 = set(arr)
    for j1 in words:
        text_after1 = normalizer.normalized_word(j1)
        lem1 = lemmatizer.lemmatize(j1).split('#')
        stem1 = stemmer.stem(j1)
        jj = 0
        if ((text_after1 in set1) or (lem1[0] in set1) or (stem1 in set1) or
(j1 in set1)):
            jj = 1
            sli.append(j1)
        text_after = []
        if (jj == 0):
            wor1 = rules_symphonious(j1)
            for x in wor1:
                norm2 = normalizer.normalized_word(x)
                lem2 = lemmatizer.lemmatize(x).split('#')
                stem2 = stemmer.stem(x)
                if (x in set1 or lem2[0] in set1 or stem2 in set1 or norm2 in
set1):
                    text_after.append(x)
            wor2 = rules_Displacement(j1)
            for x in wor2:
                norm2 = normalizer.normalized_word(x)
                lem2 = lemmatizer.lemmatize(x).split('#')
                stem2 = stemmer.stem(x)
                if (x in set1 or lem2[0] in set1 or stem2 in set1 or norm2 in
set1):
                    text_after.append(x)
            wor3 = rules_Keytransfer(j1)
            for x in wor3:
                norm2 = normalizer.normalized_word(x)
                lem2 = lemmatizer.lemmatize(x).split('#')
                stem2 = stemmer.stem(x)
                if (x in set1 or lem2[0] in set1 or stem2 in set1 or norm2 in
set1):
                    text_after.append(x)
            wor4 = rules_RepetitionCharachter(j1)
            for x in wor4:
                norm2 = normalizer.normalized_word(x)
                lem2 = lemmatizer.lemmatize(x).split('#')
                stem2 = stemmer.stem(x)
                if (x in set1 or lem2[0] in set1 or stem2 in set1 or norm2 in
set1):
                    text_after.append(x)
            wor5 = rules_Delete(j1)
            for x in wor5:
                norm2 = normalizer.normalized_word(x)
                lem2 = lemmatizer.lemmatize(x).split('#')
                stem2 = stemmer.stem(x)
                if (x in set1 or lem2[0] in set1 or stem2 in set1 or norm2 in
```

```
set1):
                    text_after.append(x)
            wor6 = rules_InsertCharctter(j1)
            for x in wor6:
                norm2 = normalizer.normalized_word(x)
                lem2 = lemmatizer.lemmatize(x).split('#')
                stem2 = stemmer.stem(x)
                if (x in set1 or lem2[0] in set1 or stem2 in set1 or norm2 in
set1):
                    text_after.append(x)
            unique_list = []
            #unique_list.append('(')
            # unique_list.append(j1)
            for x in text_after:
                # check if exists in unique_list or not
                if x not in unique_list:
                    unique_list.append(x)
            unique_list.append(j1)
            levensht_ar=[]
            for x in unique_list:
                min=100000
                for ij in set1:
                  x1=levenshtein(x,ij)
                  if(x1<min):
                      min=x1
                levensht_ar.append([x,min])
            levensht_ar1=sorted(levensht_ar, key=lambda
levensht_ar:levensht_ar[1])
            #unique_list.append(')')
            levensht_ar12=[]
            for x in levensht_ar1:
                levensht_ar12.append(x[0])
            sss =levensht_ar12[0]#'|'.join(levensht_ar12)#levensht_ar12[1]#
            sli.append(sss)
    s1 = ' '.join(sli) + "\n"
    return s1


# -----------------------------rules for symphonious-------------------------
---
def rules_symphonious(j):
    s = []
    s.append(j.replace("ه", "ح"))
    s.append(j.replace("ح", "ه"))
    s.append(j.replace("ت", "ط"))
    s.append(j.replace("ط", "ت"))
    s.append(j.replace("ع", "ا"))
    s.append(j.replace("ا", "ع"))
    s.append(j.replace("غ", "ق"))
    s.append(j.replace("ق", "غ"))
    s.append(j.replace("ث", "س"))
    s.append(j.replace("ص", "س"))
    s.append(j.replace("س", "ص"))
    s.append(j.replace("س", "ث"))
    s.append(j.replace("ض", "ز"))
    s.append(j.replace("ظ", "ز"))
    s.append(j.replace("ذ", "ز"))
    s.append(j.replace("ز", "ض"))
    s.append(j.replace("ظ", "ض"))
    s.append(j.replace("ذ", "ض"))
    s.append(j.replace("ز", "ظ"))
    s.append(j.replace("ذ", "ظ"))
    s.append(j.replace("ض", "ظ"))
    s.append(j.replace("ز", "ذ"))
    s.append(j.replace("ض", "ذ"))
```

```
    s.append(j.replace("ظ", "ذ"))
    unique_list = []
    #unique_list.append('(')
    for x in s:
        # check if exists in unique_list or not
        if x not in unique_list:
            unique_list.append(x)
    return unique_list


# -------------------------------------------Displacement Key----------------
--------
def rules_Displacement(j):
    s = []
    s.append(j.replace("گ", "ک"))
    s.append(j.replace("ک", "گ"))
    s.append(j.replace("ک", "م"))
    s.append(j.replace("م", "ک"))
    s.append(j.replace("م", "ن"))
    s.append(j.replace("ن", "م"))
    s.append(j.replace("ن", "ت"))
    s.append(j.replace("ت", "ن"))
    s.append(j.replace("ت", "ا"))
    s.append(j.replace("ا", "ت"))
    s.append(j.replace("ا", "ل"))
    s.append(j.replace("ل", "ا"))
    s.append(j.replace("ل", "ب"))
    s.append(j.replace("ب", "ل"))
    s.append(j.replace("ب", "ی"))
    s.append(j.replace("ی", "ب"))
    s.append(j.replace("ی", "س"))
    s.append(j.replace("س", "ی"))
    s.append(j.replace("س", "ش"))
    s.append(j.replace("ش", "س"))
    s.append(j.replace("پ", "چ"))
    s.append(j.replace("چ", "پ"))
    s.append(j.replace("چ", "ج"))
    s.append(j.replace("ج", "چ"))
    s.append(j.replace("ج", "ح"))
    s.append(j.replace("ح", "ج"))
    s.append(j.replace("ح", "خ"))
    s.append(j.replace("خ", "ح"))
    s.append(j.replace("خ", "ه"))
    s.append(j.replace("ه", "خ"))
    s.append(j.replace("ه", "ع"))
    s.append(j.replace("ع", "ه"))
    s.append(j.replace("ع", "غ"))
    s.append(j.replace("غ", "ع"))
    s.append(j.replace("غ", "ف"))
    s.append(j.replace("ف", "غ"))
    s.append(j.replace("ف", "ق"))
    s.append(j.replace("ق", "ف"))
    s.append(j.replace("ق", "ث"))
    s.append(j.replace("ث", "ق"))
    s.append(j.replace("ث", "ص"))
    s.append(j.replace("ص", "ث"))
    s.append(j.replace("ص", "ض"))
    s.append(j.replace("ض", "ص"))
    s.append(j.replace("و", "ئ"))
    s.append(j.replace("ئ", "و"))
    s.append(j.replace("ئ", "د"))
    s.append(j.replace("د", "ئ"))
    s.append(j.replace("د", "ذ"))
    s.append(j.replace("ذ", "د"))
    s.append(j.replace("ذ", "ر"))
```

```
    s.append(j.replace("ر", "ذ"))
    s.append(j.replace("ر", "ز"))
    s.append(j.replace("ز", "ر"))
    s.append(j.replace("ز", "ط"))
    s.append(j.replace("ط", "ز"))
    s.append(j.replace("ط", "ظ"))
    s.append(j.replace("ظ", "ط"))
    unique_list = []
    #unique_list.append('(')
    for x in s:
        # check if exists in unique_list or not
        if x not in unique_list:
            unique_list.append(x)
    return unique_list


# --------------------------------------Key transfer--------------------------
-----
def swap(s, i, j):
    if (len(s) > i and len(s) > j):
        return ''.join((s[:i], s[j], s[i + 1:j], s[i], s[j + 1:]))
    else:
        return s


def rules_Keytransfer(j):
    sli = []
    sli.append(swap(j, 0, 1))
    sli.append(swap(j, 1, 2))
    sli.append(swap(j, 2, 3))
    sli.append(swap(j, 3, 4))
    sli.append(swap(j, 4, 5))
    unique_list = []
  #  unique_list.append('(')
    for x in sli:
        # check if exists in unique_list or not
        if x not in unique_list:
            unique_list.append(x)
    return unique_list


# --------------------------------Repetition of charachter--------------------
-----------------
def Repetition(s, i):
    if (len(s) > i):
        return ''.join((s[:i], s[i], s[i], s[i + 1:]))
    else:
        return s


def rules_RepetitionCharachter(j):
    sli = []
    sli.append(Repetition(j, 0))
    sli.append(Repetition(j, 1))
    sli.append(Repetition(j, 2))
    sli.append(Repetition(j, 3))
    sli.append(Repetition(j, 4))
    unique_list = []
   # unique_list.append('(')
    for x in sli:
        # check if exists in unique_list or not
        if x not in unique_list:
            unique_list.append(x)
    return unique_list
```

```
# ---------------------------------delete of charachter-------------------------
-------------
def Delete(s, i):
    if (len(s) > i):
        return ''.join((s[:i], s[i + 1:]))
    else:
        return s


def rules_Delete(j):
    sli = []
    sli.append(Delete(j, 0))
    sli.append(Delete(j, 1))
    sli.append(Delete(j, 2))
    sli.append(Delete(j, 3))
    sli.append(Delete(j, 4))
    unique_list = []
   # unique_list.append('(')
    for x in sli:
        # check if exists in unique_list or not
        if x not in unique_list:
            unique_list.append(x)
    return unique_list


# ---------------------------------insert charctter----------------------------
---------
def insert_charctter(s, i):
    if (len(s) > i):
        switcher = {
            "ک": "گ",
            "گ": "ک",
            "ن": "ت",
            "ت": "ا",
            "ا": "ل",
            "ل": "ب",
            "ب": "ی",
            "ی": "س",
            "س": "ش",
            "ش": "س",
            "پ": "چ",
            "چ": "ج",
            "ج": "ح",
            "ح": "خ",
            "خ": "ه",
            "ه": "ع",
            "ع": "غ",
            "غ": "ف",
            "ف": "ق",
            "ق": "ث",
            "ث": "ص",
            "ص": "ض",
            "ض": "ص",
            "و": "د",
            "د": "ذ",
            "ذ": "ر",
            "ر": "ز",
            "ز": "ط",
            "ط": "ظ",
        }
        s2 = ""
        s2 = switcher.get(s[i], "")
        return ''.join((s[:i], s2, s[i], s[i + 1:]))
    else:
```

```
        return s


def insert_charctter1(s, i):
    if (len(s) > i):
        switcher = {
            "ک": "م",
            "گ": "ک",
            "م": "ک",
            "ن": "م",
            "ت": "ن",
            "ا": "ت",
            "ل": "ا",
            "ب": "ل",
            "ی": "ب",
            "س": "ی",
            "ش": "س",
            "و": "ئ",
            "ئ": "و",
            "د": "ئ",
            "ذ": "د",
            "ر": "ذ",
            "ز": "ر",
            "ط": "ز",
            "ظ": "ط",
            "پ": "چ",
            "چ": "پ",
            "ج": "چ",
            "ح": "ج",
            "خ": "ح",
            "ه": "خ",
            "ع": "ه",
            "غ": "ع",
            "ف": "غ",
            "ق": "ف",
            "ث": "ق",
            "ص": "ث",
            "ض": "ص",
        }
        s2 = ""
        s2 = switcher.get(s[i], "")
        return ''.join((s[:i], s2, s[i], s[i + 1:]))
    else:
        return s


def rules_InsertCharctter(j):
    sli = []
    sli.append(insert_charctter(j, 0))
    sli.append(insert_charctter(j, 1))
    sli.append(insert_charctter(j, 2))
    sli.append(insert_charctter(j, 3))
    sli.append(insert_charctter(j, 4))
    sli.append(insert_charctter1(j, 0))
    sli.append(insert_charctter1(j, 1))
    sli.append(insert_charctter1(j, 2))
    sli.append(insert_charctter1(j, 3))
    sli.append(insert_charctter1(j, 4))
    unique_list = []
    #unique_list.append('(')
    for x in sli:
        # check if exists in unique_list or not
        if x not in unique_list:
            unique_list.append(x)
    return unique_list
```

B. The collection of neural network codes

Table B.1. database cleaning and partitioning database

```
!pip install hazm

from google.colab import drive
drive.mount('/content/drive')

# Commented out IPython magic to ensure Python compatibility.
# %ls

import string
import re
from pickle import dump
from unicodedata import normalize
from numpy import array
from hazm import *
# load doc into memory
def load_doc(filename):
    # open the file as read only
    file = open(filename, mode='rt', encoding='utf-8')
    # read all text
    text = file.read()
    # close the file
    file.close()
    return text

# split a loaded document into sentences
def to_pairs(doc):
    lines = doc.strip().split('\n')
    pairs = [line.split('\t') for line in  lines]
    return pairs

# clean a list of lines
def clean_pairs(lines):
    cleaned = list()
    # prepare regex for char filtering
    re_print = re.compile('[^%s]' % re.escape(string.printable))
    # prepare translation table for removing punctuation
    table = str.maketrans('', '', string.punctuation)
    for pair in lines:
        clean_pair = list()
        for line in pair:
            # normalize unicode characters
            line = normalize('NFD', line).encode('ascii', 'ignore')
            line = line.decode('UTF-8')
            # tokenize on white space
            line = line.split()
            # convert to lowercase
            line = [word.lower() for word in line]
            # remove punctuation from each token
            line = [word.translate(table) for word in line]
            # remove non-printable chars form each token
            line = [re_print.sub('', w) for w in line]
            # remove tokens with numbers in them
            line = [word for word in line if word.isalpha()]
            # store as string
            clean_pair.append(' '.join(line))
        cleaned.append(clean_pair)
    return array(cleaned)
def clean_pairs1(lines):
    cleaned = list()
    for pair in lines:
```

```
        clean_pair=list()
        for line in pair:
            st1=re.sub(r'[a-zA-Z:1923456789..!؟><?123456789،]+',r'',line)
           # st1=line.replace("؟","")
            normalizer = Normalizer()
            a = normalizer.normalize(st1)
            clean_pair.append(a)
        cleaned.append(clean_pair)
    return array(  cleaned )


# save a list of clean sentences to file
def save_clean_data(sentences, filename):
    dump(sentences, open(filename, 'wb'))
    print('Saved: %s' % filename)

# load dataset
filename = '/content/drive/MyDrive/pairsiroos.txt'
doc = load_doc(filename)
# split into english-german pairs
pairs = to_pairs(doc)
#print(pairs)
# clean sentences
clean_pairs = clean_pairs1(pairs)
# save clean pairs to file
save_clean_data(clean_pairs, 'english-german.pkl')
# spot check
for i in range(100):
  print('[%s] => [%s]' % (clean_pairs[i,0], clean_pairs[i,1]))

"""# New Section"""



"""# New Section"""

from pickle import load
from pickle import dump
from numpy.random import rand
from numpy.random import shuffle

# load a clean dataset
def load_clean_sentences(filename):
   return load(open(filename, 'rb'))

# save a list of clean sentences to file
def save_clean_data(sentences, filename):
   dump(sentences, open(filename, 'wb'))
   print('Saved: %s' % filename)

# load dataset
raw_dataset = load_clean_sentences('english-german.pkl')

# reduce dataset size
n_sentences = 1000000
dataset = raw_dataset[:n_sentences, :]
# random shuffle
#shuffle(dataset)
# split into train/test
train, test = dataset[:800000], dataset[800000:1000000]
valid = raw_dataset[1000000:1190000, :]
# save
save_clean_data(dataset, 'english-german-both.pkl')
save_clean_data(train, 'english-german-train.pkl')
```

```
save_clean_data(test, 'english-german-test.pkl')
save_clean_data(valid, 'valid.pkl')
```

Table B.2. Word embedding section using fasttex

```
!pip install fasttext

from pickle import load
from numpy import array
from keras.preprocessing.text import Tokenizer
from keras.preprocessing.sequence import pad_sequences
from keras.utils import to_categorical
from keras.utils.vis_utils import plot_model
from keras.models import Sequential
from keras.layers import LSTM
from keras.layers import Bidirectional
from keras.layers import Dense
from keras.layers import Embedding
from keras.layers import RepeatVector
from keras.layers import TimeDistributed
from keras.callbacks import ModelCheckpoint
from keras.layers import Conv1D
from keras.layers import MaxPooling1D
from keras.layers import GlobalMaxPooling1D
from matplotlib import pyplot as plt
from numpy import array
from numpy import asarray
from numpy import zeros
from keras.preprocessing.text import Tokenizer
from keras.preprocessing.sequence import pad_sequences
from keras.models import Sequential
from keras.layers import Dense
from keras.layers import Flatten
from keras.layers import Embedding
from Capsule_Keras import *
from fasttext import FastText
#import fasttext
#import visualkeras
import keras
import numpy as np
#import fasttext

from sklearn.manifold import TSNE
import random


# load a clean dataset
def load_clean_sentences(filename):
    return load(open(filename, 'rb'))


# fit a tokenizer
def create_tokenizer(lines):
    tokenizer = Tokenizer()
    tokenizer.fit_on_texts(lines)
    return tokenizer


# max sentence length
def max_length(lines):
    return max(len(line.split()) for line in lines)


# encode and pad sequences
def encode_sequences(tokenizer, length, lines):
    # integer encode sequences
    X = tokenizer.texts_to_sequences(lines)
    # pad sequences with 0 values
```

```
    X = pad_sequences(X, maxlen=length, padding='post')
    return X


# one hot encode target sequence
def encode_output(sequences, vocab_size):
    ylist = list()
    for sequence in sequences:
        encoded = to_categorical(sequence, num_classes=vocab_size)
        ylist.append(encoded)
    y = array(ylist)
    y = y.reshape(sequences.shape[0], sequences.shape[1], vocab_size)
    return y


def pre_train_glove(vocab_size, word_index):
    embeddings_index = dict()
    f = open('../glove_data/glove.6B/glove.6B.100d.txt')
    for line in f:
        values = line.split()
        word = values[0]
        coefs = asarray(values[1:], dtype='float32')
        embeddings_index[word] = coefs
    f.close()
    print('Loaded %s word vectors.' % len(embeddings_index))
    # create a weight matrix for words in training docs
    embedding_matrix = zeros((vocab_size, 100))
    for word, i in word_index.items():
        embedding_vector = embeddings_index.get(word)
        if embedding_vector is not None:
            embedding_matrix[i] = embedding_vector
    return embedding_matrix


def pre_train_fasttext(words):

    fastText_model_path = '/content/drive/MyDrive/FA.bin'
    model = FastText.load_model(fastText_model_path)
    embedding_matrix = zeros((len(words)+1, 300))
    for i in range(0,len(words)-1):
       embedding_matrix[i] = model.get_word_vector(words[i])

    return embedding_matrix
# define NMT model
def define_model(src_vocab, tar_vocab, src_timesteps, tar_timesteps,
n_units,embedding_matrix):
    model = Sequential()
  # model.add(Embedding(src_vocab, n_units, input_length=src_timesteps))
    model.add(Embedding(src_vocab,
300,weights=[embedding_matrix],input_length=src_timesteps,trainable=False))
   # model.add(Conv1D(4 * n_units, 3, activation='relu', padding='same'))
    #model.add(MaxPooling1D(pool_size=2, padding='same'))
    #model.add(Capsule(num_capsule=10, dim_capsule=16, routings=3,
share_weights=True))
    model.add(Bidirectional(LSTM(n_units)))
    model.add(RepeatVector(tar_timesteps))
    model.add(Bidirectional(LSTM(n_units, return_sequences=True)))
    model.add(TimeDistributed(Dense(tar_vocab, activation='softmax')))
    # visualkeras.layered_view(model, to_file='output.png')
    return model


# load datasets
dataset = load_clean_sentences('english-german-both.pkl')
```

```
# prepare english tokenizer
eng_tokenizer = create_tokenizer(dataset[:, 0])
#print(eng_tokenizer.word_index.keys())
#print(eng_tokenizer.word_index)
eng_vocab_size = len(eng_tokenizer.word_index) + 1
eng_length = max_length(dataset[:, 0])
#print('English Vocabulary Size: %d' % eng_vocab_size)
#print('English Max Length: %d' % (eng_length))
# prepare german tokenizer
ger_tokenizer = create_tokenizer(dataset[:, 1])
ger_vocab_size = len(ger_tokenizer.word_index) + 1
ger_length = max_length(dataset[:, 1])
#print(ger_tokenizer.word_index)
wordFastTet=[]
for key in ger_tokenizer.word_index:
    wordFastTet.append(key)

#print(ger_tokenizer.word_index)
print('German Vocabulary Size: %d' % ger_vocab_size)
print('German Max Length: %d' % (ger_length))
embedding_matrix=pre_train_fasttext(wordFastTet)
np.savetxt("embedding_matrix.txt",embedding_matrix)
```

Table B.3. Network architecture construction section and its training

```
#y = np.loadtxt('embedding_matrix.txt')
#print(y[3200])
!pip install XlsxWriter

from pickle import load
from numpy import array
from keras.preprocessing.text import Tokenizer
from keras.preprocessing.sequence import pad_sequences
from keras.utils import to_categorical
from keras.utils.vis_utils import plot_model
from keras.models import Sequential
from keras.layers import LSTM
from keras.layers import Bidirectional
from keras.layers import Dense
from keras.layers import Embedding
from keras.layers import RepeatVector
from keras.layers import TimeDistributed
from keras.callbacks import ModelCheckpoint
from keras.layers import Conv1D
from keras.layers import MaxPooling1D
from keras.layers import GlobalMaxPooling1D
from matplotlib import pyplot as plt
from keras.layers import Dropout
from numpy import array
from numpy import asarray
from numpy import zeros
from keras.preprocessing.text import Tokenizer
from keras.preprocessing.sequence import pad_sequences
from keras.models import Sequential
from keras.layers import Dense
from keras.layers import Flatten
from keras.layers import Embedding
from keras.layers import BatchNormalization
from Capsule_Keras import *
import numpy as np
import xlwt
from xlwt import Workbook
import pandas as pd
import pandas as pd
import numpy as np
from keras.callbacks import EarlyStopping
#import fasttext
#import visualkeras
#import fasttext

from sklearn.manifold import TSNE
import random


# load a clean dataset
def load_clean_sentences(filename):
    return load(open(filename, 'rb'))


# fit a tokenizer
def create_tokenizer(lines):
    tokenizer = Tokenizer()
    tokenizer.fit_on_texts(lines)
    return tokenizer


# max sentence length
def max_length(lines):
```

```python
    return max(len(line.split()) for line in lines)


# encode and pad sequences
def encode_sequences(tokenizer, length, lines):
    # integer encode sequences
    X = tokenizer.texts_to_sequences(lines)
    # pad sequences with 0 values
    X = pad_sequences(X, maxlen=length, padding='post')
    return X


# one hot encode target sequence
def encode_output(sequences, vocab_size):
    ylist = list()
    for sequence in sequences:
      #  encoded = to_categorical(sequence, num_classes=vocab_size)
        ylist.append(sequence)
    y = array(ylist)
    y = y.reshape(sequences.shape[0], sequences.shape[1])
    return y

# define NMT model
def define_model(src_vocab, tar_vocab, src_timesteps, tar_timesteps,
n_units,embedding_matrix):


   # model.add(Embedding(src_vocab,
300,weights=[embedding_matrix],input_length=src_timesteps,trainable=True))
   # model.add(Conv1D(4 * n_units, 3, activation='relu', padding='same'))
    #model.add(MaxPooling1D(pool_size=2, padding='same'))
  #  model.add(Capsule(num_capsule=10, dim_capsule=16, routings=3,
share_weights=True))
    #model.add(Bidirectional(LSTM(n_units)))
    model = Sequential()
   # model.add(Embedding(src_vocab, 100, input_length=src_timesteps,
mask_zero=True))
    model.add(Embedding(src_vocab,
300,weights=[embedding_matrix],input_length=src_timesteps,trainable=True))
   # model.add(Conv1D(100, 3, activation='relu', padding='same'))
   # model.add(MaxPooling1D(pool_size=2, padding='same'))
    #model.add(Capsule(num_capsule=50, dim_capsule=50, routings=3,
share_weights=True))
    model.add(Bidirectional(LSTM(1000,dropout=0.5)))
    model.add(RepeatVector(tar_timesteps))
    #model.add(Bidirectional(LSTM(n_units,dropout=0.5)))
   # model.add(RepeatVector(tar_timesteps))
    model.add(Bidirectional(LSTM(1000, return_sequences=True, dropout=0.5)))
    model.add(TimeDistributed(Dense(tar_vocab, activation='softmax')))
  #  visualkeras.layered_view(model)
    return model



# load datasets
dataset = load_clean_sentences('english-german-both.pkl')
train = load_clean_sentences('english-german-train.pkl')
test = load_clean_sentences('english-german-test.pkl')

# prepare english tokenizer
eng_tokenizer = create_tokenizer(dataset[:, 0])
#print(eng_tokenizer.word_index.keys())
#print(eng_tokenizer.word_index)
eng_vocab_size = len(eng_tokenizer.word_index) + 1
eng_length = max_length(dataset[:, 0])
```

```
print('English Vocabulary Size: %d' % eng_vocab_size)
print('English Max Length: %d' % (eng_length))
# prepare german tokenizer
ger_tokenizer = create_tokenizer(dataset[:, 1])
ger_vocab_size = len(ger_tokenizer.word_index) + 1
ger_length = max_length(dataset[:, 1])
#print(ger_tokenizer.word_index)


#print(ger_tokenizer.word_index)
print('German Vocabulary Size: %d' % ger_vocab_size)
print('German Max Length: %d' % (ger_length))

# prepare training data
trainX = encode_sequences(ger_tokenizer, ger_length, train[:, 1])
trainY = encode_sequences( eng_tokenizer, eng_length, train[:, 0])
trainY = encode_output(trainY, eng_vocab_size)
# prepare validation data
testX = encode_sequences(ger_tokenizer, ger_length, test[:, 1])
testY = encode_sequences( eng_tokenizer, eng_length, test[:, 0])
testY = encode_output(testY, eng_vocab_size)
print('finish stup')
# embedding_matrix=pre_train_glove(ger_vocab_size,word_index)
embedding_matrix = np.loadtxt('embedding_matrix.txt')
# define model
hi=[]
wb = Workbook()
www='/content/drive/MyDrive/'+'data.xlsx'
writer1=pd.ExcelWriter(www)
df1 = pd.DataFrame()
df1.to_excel(writer1, sheet_name='x1')
writer1.save()
writer1.close()
for i in range(1,2):
 writer = pd.ExcelWriter(www, engine='openpyxl', mode='a')
 model = define_model(ger_vocab_size, eng_vocab_size, ger_length,
eng_length,1000,embedding_matrix)
 model.compile(optimizer='rmsprop', loss='sparse_categorical_crossentropy',
metrics=['acc'])
# summarize defined model
 print(model.summary())
# plot_model(model, to_file='model.png', show_shapes=True)
# keras.utils.plot_model(model, "m1.png", show_shapes=True)

# fit model
 filename = '/content/drive/MyDrive/'+'model'+str(i)+'.h5'
 checkpoint = ModelCheckpoint(filename, monitor='acc', verbose=1,
save_best_only=True, mode='max')
 es = EarlyStopping(monitor='val_loss', mode='min', verbose=1, patience=100)
 history = model.fit(trainX,trainY, epochs=2000, batch_size=64,
validation_data=(testX, testY), callbacks=[checkpoint,es],
                    verbose=2)

 #strcom=str(i)+'.txt'
 #sheet1 = wb.add_sheet(strcom)
 #w2 = open(strcom,'w', encoding='utf-8')
 df = pd.DataFrame(history.history)
 df.to_excel(writer, sheet_name=str(i))
 #for i in range(len(history.history['acc'])):
  #w2.write(str(history.history['acc'][i])+'
'+str(history.history['val_acc'][i])+' '+str(history.history['loss'][i])+'
'+str(history.history['val_loss'][i])+'\n')
  #sheet1.write(0, 0, 'ISBT DEHRADUN')
 #w2.close()
 plt.plot(history.history['acc'])
```

```
plt.plot(history.history['val_acc'])
plt.title('model accuracy')
plt.ylabel('accuracy')
plt.xlabel('epoch')
plt.legend(['train', 'val'], loc='upper left')
str1='accuracy'+str(i)+'.png'
str2='loss'+str(i)+'.png'
plt.savefig(str1)
plt.show()
plt.plot(history.history['loss'])
plt.plot(history.history['val_loss'])
plt.title('model loss')
plt.ylabel('loss')
plt.xlabel('epoch')
plt.legend(['train', 'val'], loc='upper left')
plt.savefig(str2)
plt.show()
writer.save()
writer.close()
```

Table B.4. network prediction

```
from pickle import load


from numpy import argmax
from keras.preprocessing.text import Tokenizer
from keras.preprocessing.sequence import pad_sequences
from keras.models import load_model
from nltk.translate.bleu_score import corpus_bleu
from keras.models import load_model
from Capsule_Keras import *
# load a clean dataset
def load_clean_sentences(filename):
   return load(open(filename, 'rb'))

# fit a tokenizer
def create_tokenizer(lines):
   tokenizer = Tokenizer()
   tokenizer.fit_on_texts(lines)
   return tokenizer

# max sentence length
def max_length(lines):
   return max(len(line.split()) for line in lines)

# encode and pad sequences
def encode_sequences(tokenizer, length, lines):
   # integer encode sequences
   X = tokenizer.texts_to_sequences(lines)
   # pad sequences with 0 values
   X = pad_sequences(X, maxlen=length, padding='post')
   return X

# map an integer to a word
def word_for_id(integer, tokenizer):
   for word, index in tokenizer.word_index.items():
      if index == integer:
         return word
   return None

# generate target given source sequence
def predict_sequence(model, tokenizer, source):
   #print(source)
   prediction = model.predict(source, verbose=0)[0]
   integers = [argmax(vector) for vector in prediction]
   target = list()
   for i in integers:
      word = word_for_id(i, tokenizer)

      if word is None:
         break
      target.append(word)
   return ' '.join(target)

# evaluate the skill of the model
def evaluate_model(model, tokenizer, sources, raw_dataset):
    w1 = open("/content/drive/MyDrive/formann.txt","w", encoding='utf-8')
    w2 = open("/content/drive/MyDrive/inform1.txt","w", encoding='utf-8')
    w3 = open("/content/drive/MyDrive/formtrue.txt","w", encoding='utf-8')
    actual, predicted = list(), list()
    for i, source in enumerate(sources):
        # translate encoded source text
        source = source.reshape((1, source.shape[0]))
```

```
        translation = predict_sequence(model, eng_tokenizer, source)
        # print(translation)
        # print(raw_dataset[i])
        raw_target, raw_src= raw_dataset[i]

        #print('src=[%s], target=[%s], predicted=[%s]' % (raw_src, raw_target, 
translation))
        w1.write(translation+'\n')
        w2.write(raw_src.split('\n')[0]+'\n')
        w3.write(raw_target+'\n')
       # actual.append([raw_target.split()])
      #  predicted.append(translation.split())
    w1.close()
    w2.close()
    w3.close()
    # calculate BLEU score
    #print('BLEU-1: %f' % corpus_bleu(actual, predicted, weights=(1.0, 0, 0, 
0)))
    #print('BLEU-2: %f' % corpus_bleu(actual, predicted, weights=(0.5, 0.5, 0, 
0)))
    #print('BLEU-3: %f' % corpus_bleu(actual, predicted, weights=(0.3, 0.3, 
0.3, 0)))
    #print('BLEU-4: %f' % corpus_bleu(actual, predicted, weights=(0.25, 0.25, 
0.25, 0.25)))
# evaluate the skill of the model
def evaluate_model1(model, tokenizer, sources, raw_dataset):
   actual, predicted = list(), list()
   for i, source in enumerate(sources):
      # translate encoded source text
      source = source.reshape((1, source.shape[0]))
      translation = predict_sequence(model, eng_tokenizer, source)
      raw_src = raw_dataset[i]
      #print(translation)
      #print(raw_dataset[i])
      if i < 10:
         print('src=[%s], predicted=[%s]' % (raw_src, translation))
      predicted.append(translation.split())
# load datasets
dataset = load_clean_sentences('english-german-both.pkl')
train = load_clean_sentences('english-german-train.pkl')
test = load_clean_sentences('english-german-test.pkl')
vtest = load_clean_sentences('valid.pkl')
# prepare english tokenizer
eng_tokenizer = create_tokenizer(dataset[:, 0])
eng_vocab_size = len(eng_tokenizer.word_index) + 1
eng_length = max_length(dataset[:, 0])
# prepare german tokenizer
ger_tokenizer = create_tokenizer(dataset[:, 1])
ger_vocab_size = len(ger_tokenizer.word_index) + 1
ger_length = max_length(dataset[:, 1])
# prepare data
#trainX = encode_sequences(ger_tokenizer, ger_length, train[:, 1])
#testX = encode_sequences(ger_tokenizer, ger_length, test[:, 1])
vtestX = encode_sequences(ger_tokenizer, ger_length, vtest[:, 1])
a=list()
a.append("است سابت من زندگی حمه")
a.append("هسمت قریب شره این در")
a.append("حملح بدح ادامح")
x1= encode_sequences(ger_tokenizer, ger_length,a)

# load model
r1 = open("/content/drive/MyDrive/informalAnn.txt", encoding='utf-8')
f1=r1.readlines()
validation=list()
for k in f1:
```

```
  validation.append(k)
r1.close()
validationx= encode_sequences(ger_tokenizer, ger_length,validation)
modeltrain='/content/drive/MyDrive/bi1000.h5'
model = load_model(modeltrain, custom_objects={'Capsule': Capsule})
#model = load_model('model.h5')
# test on some training sequences
#evaluate_model1(model, eng_tokenizer, x1, a)
print('train')
#evaluate_model(model, eng_tokenizer, validationx, validation)
evaluate_model(model, eng_tokenizer, vtestX, vtest)
#evaluate_model(model, eng_tokenizer, trainX, train)
# test on some test sequences
print('test')
#evaluate_model(model, eng_tokenizer, testX, test)
```

Table B.5. Evaluation section code and obtaining evaluation quantities

```
from nltk.translate.bleu_score import corpus_bleu
import numpy as np
from sklearn.metrics import precision_recall_fscore_support
from sklearn.metrics import accuracy_score
r1 = open("C:\\Users\\saman\\PycharmProjects\\untitled\\informalAnn.txt",
encoding='utf-8')
r2 = open("C:\\Users\\saman\\PycharmProjects\\untitled\\formalAnn2.txt",
encoding='utf-8')
r3=open("C:\\Users\\saman\\PycharmProjects\\untitled\\formaltrue.txt",
encoding='utf-8')
f1=r1.readlines()
f2=r2.readlines()
f3=r3.readlines()
tp=0
tn=0
fp=0
fn=0
l1=0
for i in range(0,len(f1)):
    a1=f1[i].split('\n')[0].split(' ')
    a2=f2[i].split('\n')[0].split(' ')
    a3=f3[i].split('\n')[0].split(' ')
    l1=l1+len(a3)
    max1=min(len(a3),len(a2),len(a1))
   # while len(a3)<max1:
   #     a3.append(' ')
    #while len(a2)<max1:
    #    a2.append(' ')
   # while len(a1)<max1:
      #  a1.append(' ')
    for j in range(0,max1):
        if(a3[j]==a1[j] and a3[j]==a2[j]):
            tn=tn+1
        elif (a3[j]==a1[j] and a3[j]!=a2[j]):
            fn=fn+1

        elif (a3[j] != a1[j] and a3[j] == a2[j]):
             tp=tp+1
        elif (a3[j] != a1[j] and a3[j] != a2[j]):
            fp=fp+1
print('tp: %f' % tp)
print('tn: %f' % tn)
print('fp: %f' % fp)
print('fn: %f' % fn)
ftotal=tp+tn+fp+fn
print('toralTpFpTnFn: %f' % ftotal)
print('totalwords: %f' % l1)
accuracy=(tp+tn)/(tp+tn+fp+fn)
perection=(tp)/(tp+fp)
recall=(tp)/(tp+fn)
f_meaure=(2*perection*recall)/(perection+recall)
print("accuracy:")
print(accuracy)
print("perection:")
print(perection)
print("recall:")
print(recall)
print("f_meaure:")
print(f_meaure)
actual, predicted = list(), list()
actual1=list()
for i in range(0,len(f1)-1):
            actual.append([f3[i].split('\n')[0].split(' ')])
```

```
            predicted.append(f2[i].split('\n')[0].split(' '))
            actual1.append(f3[i].split('\n')[0].split(' '))
print('BLEU-1: %f' % corpus_bleu(actual, predicted, weights=(1.0, 0, 0, 0)))
print('BLEU-2: %f' % corpus_bleu(actual, predicted, weights=(0, 1.0, 0, 0)))
print('BLEU-3: %f' % corpus_bleu(actual, predicted, weights=(0, 0,1.0, 0)))
print('BLEU-4: %f' % corpus_bleu(actual, predicted, weights=(0.25, 0.25, 0.25,
0.25)))
print('BLEUTotal: %f' % corpus_bleu(actual, predicted))
array1=[]
actual2=''
predicted2=''
for i in range(0,len(actual1)):
    max2=min(len(actual1[i]),len(predicted[i]))
    while len(actual1[i])>max2:
        actual1[i].pop()
    while len(predicted[i])>max2:
        predicted[i].pop()
    for ii in range(0,len(actual1[i])):
        actual2=actual2 +' ' +actual1[i][ii]
        predicted2=predicted2+' '+predicted[i][ii]
    a=precision_recall_fscore_support(actual1[i], predicted[i],
average='micro')
    array1.append(a)
kk=0
per=0
re=0
f=0
print("test")
print(len(array1))
for i in range(500):
    #print(array1[i])
    per=per+array1[i][0]
    re = re+array1[i][1]
    f=f+array1[i][2]
    kk=kk+1
print(per/kk)
print(re/kk)
print(f/kk)
print(precision_recall_fscore_support(actual2.split(' '),predicted2.split('
'),average='micro'))
print( accuracy_score(actual2.split(' '), predicted2.split(' ')))
```
